# Catecholamine exposure and the gut microbiota in obstructive sleep apnea

**DOI:** 10.7717/peerj.19203

**Published:** 2025-04-14

**Authors:** Joe Alcock, Dongdong Lin, Prashanth Setty, Lee K. Brown, Armand E.K. Dichosa, Benjamin J. Burnett, Cliff S. Han, Henry C. Lin

**Affiliations:** 1 Department of Emergency Medicine, University of New Mexico Health Sciences Center, Albuquerque, NM, United States of America; 2 EMD Serono, Inc, Boston, MA, United States of America; 3 Applied Biomedical, Inc, Placentia, CA, United States of America; 4 New Mexico VA Health Care System, Albuquerque, NM, United States of America; 5 Division of Pulmonary, Critical Care and Sleep Medicine, Department of Internal Medicine, University of New Mexico, Albuquerque, NM, United States of America; 6 Los Alamos National Laboratory, Los Alamos, NM, United States of America; 7 Ascent Healthcare, Beaverton, OR, United States of America; 8 Knoze Jr, Los Alamos, NM, United States of America; 9 Division of Gastroenterology, Department of Internal Medicine, University of New Mexico, Albuquerque, NM, United States of America; 10 Section of Gastroenterology, New Mexico VA Health Care System, Albuquerque, NM, United States of America

**Keywords:** Sympathetic nervous system, Circadian rhythm, Microbiome, Obstructive sleep apnea, Norepinephrine, Sleep disorder

## Abstract

Patients with obstructive sleep apnea (OSA) have increased mortality from chronic inflammatory and cardiovascular diseases. Excess catecholamine exposure contributes to the disease associations of OSA, but the underlying mechanism is unknown. This study tested the hypothesis that increased catecholamine exposure is associated with Enterobacteriaceae abundance in OSA. We compared urinary norepinephrine and the fecal microbiota in 24 patients with OSA and 23 controls. Urinary norepinephrine was elevated in OSA patients, consistent with increased sympathetic activation in those patients. OSA patients did not show changes in the community structure of the microbiome or in Enterobacteriaceae abundance compared to controls. Longitudinal changes in Enterobacteriaceae abundance in OSA patients were significantly associated with within-subject changes in norepinephrine, but this association was absent in controls. These results provide a preliminary association between norepinephrine exposure and Enterobacteriaceae in patients with disordered sleep.

## Introduction

Obstructive sleep apnea (OSA) is a condition of fragmented sleep caused by upper airway obstruction that has increased in prevalence over recent decades (Peppard et al., 2013). Moderate to severe OSA, defined as over 15 apneic or hypopneic episodes per hour, is estimated to occur in up to 17% of men and 9% of women (Peppard et al., 2013). OSA increases the risk of all-cause mortality (Yaggi et al., 2005) and is linked to chronic inflammatory diseases (Hoevenaar-Blom et al., 2011; Cappuccio et al., 2010).

Recent work suggests that circadian disruption and disordered sleep have an adverse effect on the microbiome. Sleep fragmentation and intermittent hypoxia can change the composition of the gut microbiota (Moreno-Indias et al., 2015; Benedict et al., 2016). Experimental sleep deprivation in rodents can impair intestinal barrier function (Everson, 2005) and cause intestinal inflammation (Everson, Thalacker & Hogg, 2008; Tang et al., 2009). Intermittent hypoxia, a hallmark of OSA, has been shown to alter the function and composition of the gut microbiota in mice (Tripathi et al., 2018).

Studies of patients with OSA indicate that the gut microbiota may play a role in its complications, such as insulin resistance and hypertension (Poroyko et al., 2016; Durgan et al., 2016). Increased intestinal permeability can occur in pediatric OSA (Kheirandish-Gozal et al., 2014) and in adult OSA along with changes to the gut microbiota (Li et al., 2023). Altered gut microbiota may exacerbate low grade systemic inflammation in OSA. For example, OSA has been associated with increased pro-inflammatory Alistipes in the gut (Wang et al., 2022). Li et al. (2023) showed an association between pro-inflammatory *Fusobacterium* and hypoxia along with a reduction of short chain fatty acid-producing bacteria in adults with OSA.

Catecholamine exposure is a potential link between hypoxia in OSA and the gut microbiota. OSA involves upper airway obstruction resulting in interrupted ventilation and hypoxia during sleep. Hypoxia, in turn, results in nocturnal arousals that fragment the normal architecture of sleep. These arousals are accompanied by adrenal production of stress catecholamines, including norepinephrine and epinephrine. Intermittent hypoxia and disordered sleep have been shown to result in increased circulating catecholamines in preclinical studies and in patients with OSA (Elmasry et al., 2002; Cetin-Atalay et al., 2021). Plasma and urinary norepinephrine are elevated in patients with OSA because of increased sympathetic nerve activity during the awake period and nocturnal surges of sympathetic activity following apneic events (Somers et al., 1995; Elmasry et al., 2002). Elevated catecholamines in OSA has potential relevance to the microbiome because catecholamines were shown to significantly increase the growth of gram-negative pathogens and pathobionts in preclinical studies. Norepinephrine, in particular, has been reported to increase the growth of *Desulfovibrio*, *Escherichia*, *Klebsiella*, *Yersinia*, and *Salmonella* (Lyte & Ernst, 1992; Belay & Sonnenfeld, 2002; Belay et al., 2003; Freestone, Haigh & Lyte, 2007; Coffman et al., 2022). These findings highlight the untested possibility that catecholamines change the gut microbiota in obstructive sleep apnea.

It is unknown whether elevated catecholamines might affect the growth of gram-negative pathogens in OSA. However, several groups have reported altered gut microbiota in OSA involving these bacteria. Ko et al. (2019) reported an overgrowth of proinflammatory gram negative enteric pathogens, including those in the family Enterobacteriaceae*.* An increase in Enterobacter was observed in pediatric OSA patients with high apnea-hypoxia indices (Chuang et al., 2024). Increased LPS-binding protein—serving as a surrogate marker of gram-negative endotoxemia—was documented in pediatric OSA (Kheirandish-Gozal et al., 2014). In the present study, CPAP is postulated to affect systemic catecholamines and was leveraged to test the effects of catecholamines on the gut microbiota. The aim of this pilot study was to examine whether norepinephrine, which is known to be elevated in patients with obstructive sleep apnea, is associated with changes in the gut microbiota.

To the best of our knowledge, no published study has investigated a potential effect of systemic catecholamine exposure on gut microbiota in patients with OSA. The present study was undertaken to examine whether changes in catecholamine exposure, influenced by OSA disease status and CPAP therapy, has an influence the diversity of the gut microbiota. We tested the hypothesis that patients with obstructive sleep apnea have catecholamine-driven changes in the community structure of the gut microbiota. OSA patients were hypothesized to have increased catecholamines and increased density of intestinal Enterobacteriaceae, a bacterial group that contains many important gram-negative pathogens. Because OSA treatment with continuous positive airway pressure (CPAP) can limit apneic episodes and reduce norepinephrine levels (Somers et al., 1995; Sukegawa et al., 2005; Phillips et al., 2007), we hypothesized that successful treatment with CPAP would result in lower catecholamine exposure and decreased density of Enterobacteriaceae.

## Materials & Methods

Twenty four patients with obstructive sleep apnea were recruited at the University of New Mexico Sleep Disorders Center in 2012-2013. Twenty three controls without sleep disorders were recruited from the UNM Clinical Translational Science Center subject database. We compared the intestinal microbiota and urinary catecholamines in newly-diagnosed OSA patients and control subjects. OSA patients and control subjects who completed the protocol underwent fecal and urinary catecholamine sampling twice, separated by a minimum of four weeks, to assess the effect of treatment with continuous positive airway pressure on OSA patients.

Subjects were eligible if newly diagnosed with obstructive sleep apnea of moderate severity or greater, defined by an apnea hypopnea index >15 using American Academy of Sleep Medicine criteria, age of 21 years of age or older and eligible for CPAP treatment. Exclusion criteria included use within 4 weeks of antibiotics, probiotic supplements, corticosteroids, iron, proton pump inhibitors, or alcoholic drinks in excess of 14 per week, and pregnancy. These were obtained by self-report. Controls were also excluded if they had self-reported sleep disorders. Height and weight were measured without shoes on calibrated scales. Body mass index was calculated as weight in kilograms divided by height in meters squared.

The Albuquerque VA Research and Development Committee and the University of New Mexico Human Research Protections Office approved the protocol and written informed consent was obtained from all subjects (HRPO #11-226).

### Sample collection

Subjects completed a home collection of urine and stool which was refrigerated and delivered to the researchers on the day of collection. For patients with OSA, second samples were collected after >4 weeks of CPAP. Because Phillips et al. (2007) reported an effect of CPAP on urinary norepinephrine within one week, we assumed that two months would be sufficient time to influence sympathetic activation and catecholamine exposure. Electronic usage records allowed measurement of CPAP adherence which was defined as 70% or greater nightly CPAP usage with at least 4 h use per night and/or 5 h or more median daily use. For control subjects, 2nd samples were collected 4–8 weeks after 1st collections. Urine was collected over a 24-hour period and urine samples were sent to ARUP Reference Laboratories Salt Lake City, Utah, for catecholamine analysis on LC-MS/MS, methods described in Kushnir et al. (2002). Stool specimens were stored at −80 °C.

### DNA extraction

Samples were then thawed for DNA extraction from which a 0.18–0.22 g stool aliquot was transferred to a DNA/RNA-free sterile tube, 200 µl of ASL buffer was added, and the sample was vortexed until homogenization. The homogenate was then used for DNA extraction with a QIAmp DNA Stool Mini Kit *via* protocol described by Qiagen (Hilden, Germany). Extracted DNA was eluted in AE buffer and quantified using the Nanovue Plus spectrophotometer and then stored at −20 °C.

### Fecal microbiota analysis by quantitative PCR

PCR primers specific for the 16S rRNA gene were used to target Enterobacteriaceae and Universal (total) bacteria (Table S1). PCR amplification and detection were performed with a Mastercycler Realplex real-time PCR system (Eppendorf AG, Hamburg, Germany). Each plate cell contained a four µl reaction mixture which consisted of SYBR Green PCR Master Mix (SuperArray Bioscience, Foster City, CA), primers, and 100 ng of template DNA. Each PCR reaction was performed in duplicate; the mean of the duplicate cycle threshold (Ct) values was used. Bacterial gene abundance in each sample was calculated by comparing the Ct values obtained from the standard curves. Standard curves were created using serial tenfold dilution of pure cultures of DNA, corresponding to 10–1012 cells from the culture collection as determined by microscopy counts. Enterobacteriacae abundance was expressed as the proportion of log Enterobacteriaceae gene copies/ log Universal gene copies.

### Illumina sequencing

The Bioscience Division at Los Alamos National laboratory (Los Alamos, NM) performed deep 16S rRNA gene sequencing of the microbiome. The sequencing used an Illumina MiSeq platform based on differences in the V4 region of the 16S ribosomal DNA of bacteria (Illumina, San Diego, CA). The V4 variable region of the 16S rRNA gene was amplified with a NEXTflex 16S V4 Amplicon-Seq Kit (with 96 barcodes) (BIOO Scientific, Austin, TX) following the manufacturer’s instructions. Each sample was PCR amplified with differently barcoded V4 fusion primers. In total, 50 ng DNA was used from each sample. The amplified DNAs were verified on a gel and pooled at an equal molar ratio. The pooled DNA samples were purified with Agencourt AMPure beads. Pooled samples containing 16S V4 enriched, amplified, barcoded samples were loaded into the reagent cartridge and then onto the instrument. Amplicons were sequenced for 250 cycles with standard primers designed for paired-end sequencing.

This process yielded 18,712,064 sequences that were used in the analysis to identify operational taxonomic units (OTUs). OTUs are the functional equivalent of species present in the microbiome and were clustered at 97% identical level using closed reference OTU picking, searched against the SILVA reference database of 16S rDNA bacterial sequences (http://www.ncbi.nlm.nih.gov/pubmed/23193283). Beta diversity (weighted unifrac and unweighted unifrac distance matrices) were applied to resulting taxonomies (Lozupone et al., 2011) to estimate phylogenetic distances between microbial communities. Bray-curtis was calculated as a non-parametric metric of beta diversity. Shannon index and chao1 were used to measure alpha diversity. Sequence data for this project are publicly accessible at the NCBI sequence read archive, project number SRP077632.

### Fecal calprotectin

We used the Hycult HK325 human calprotectin ELISA kit, Hycult Biotechnology, to measure calprotectin as a measure of intestinal inflammation. 100 mg from each fecal sample was added to 4.9 mL extraction buffer (0.1 M Tris, 0.15 M NaCl, 1.0 M urea, 10 mM CaCl2⋅2H2O, 0.1 M citric acid, 0.5% bovine serum albumin, 0.25 mM thimerosal), according the manufacturer’s instructions. Samples were vortexed and the supernatant was aliquoted and stored at −20 °C. Calprotectin concentration was measured in lysate by ELISA (lower detection limit, 625 ng/mL). Fecal calprotectin concentration is given in micrograms of calprotectin per gram of feces. Analysis was performed on the mean of two measurements taken per sample.

### Statistical tests

Normally distributed variables, including age, BMI, and urine norepinephrine concentration, were subjected to t-tests (two-sample and paired). Simple linear regression was used to probe the relationship between norepinephrine and other variables on fecal Enterobacteriaceae abundance. Longitudinal observations that were clustered by subject (pre- and post-treatment in OSA patients; and 1st and 2nd specimen collections in controls) were analyzed using multilevel mixed-effects linear regression (xtmixed procedure). Unweighted and weighted unifrac testing tested for differences based on presence/absence and abundance of microbial taxa in the fecal microbiome. We used the PERMANOVA method and controlled for age, sex and BMI to measure between-sample differences in beta diversity. Additional statistical tests were performed using STATA (version 11.2).

## Results

**Figure 1:**
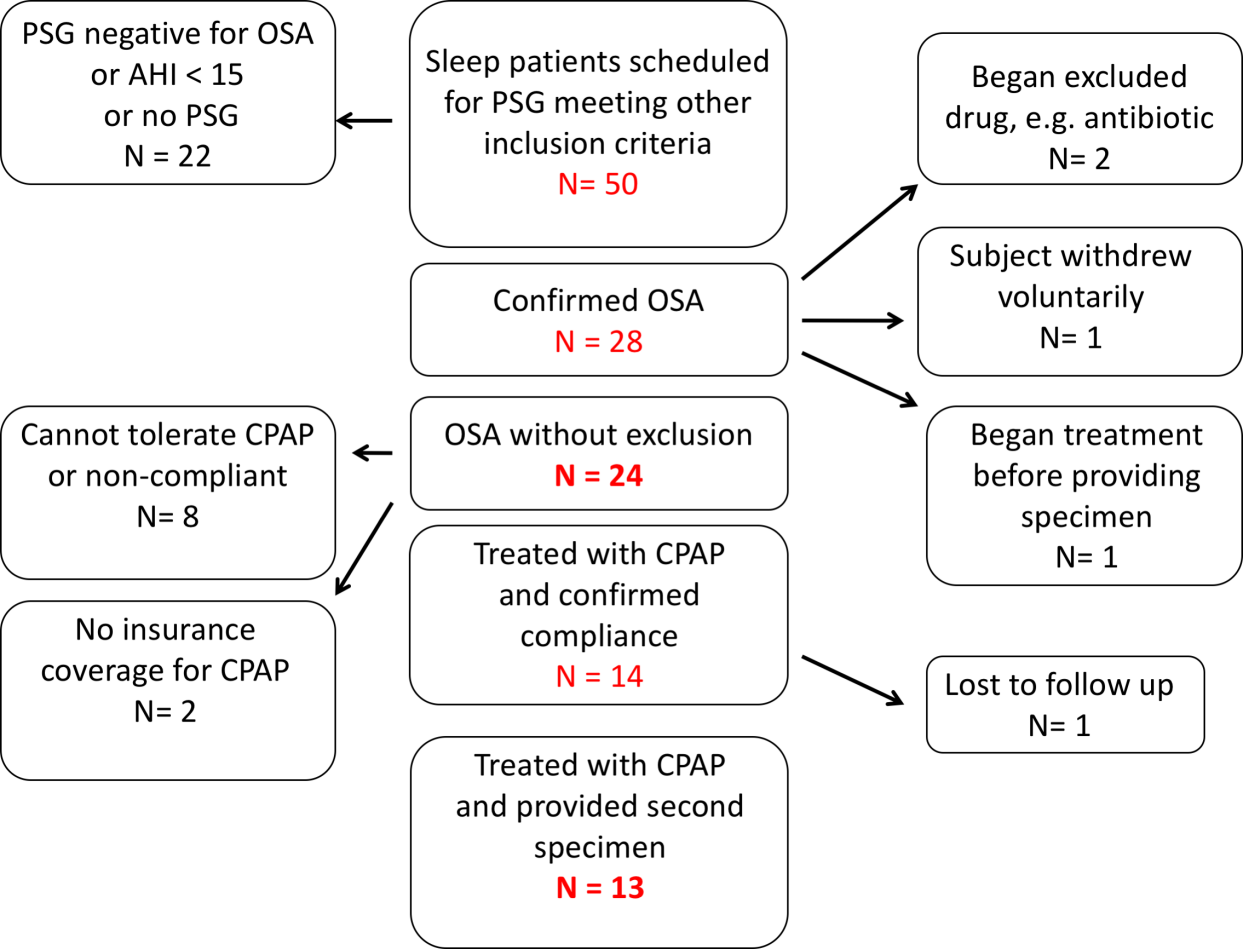
Recruitment of patients with obstructive sleep apnea. Of 50 subjects enrolled, 24 met all inclusion and exclusion criteria and provided a first sample of stool and urine. Thirteen enrolled subjects were treated with continuous positive airway pressure and provided samples for second fecal bacteriological and urinary catecholamine analysis.

Fifty patients at the UNMH sleep disorders center with suspected OSA were screened with a diagnostic polysomnogram (Fig. 1). Of these, we enrolled 24 OSA patients who met all inclusion and exclusion criteria. Thirteen OSA patients provided both pre-treatment (1st collection) and post-treatment with CPAP (2nd collection) fecal and urine specimens (Fig. 1).

The sample was predominantly middle aged and the age of OSA patients did not differ significantly from that of controls (Table 1). Females outnumbered males in both groups. Subjects with OSA had higher BMIs than controls. Patients with OSA had a mean of 63 apneic or hypopneic events per hour.

**Table 1:** Subject characteristics.

Sample characteristics			
	OSA Patient (*n* = 24)	Control (*n* = 23)	*t*-test
Age (mean ± SE)	47.8 ± 2	44.3 ± 2.4	*p* = 0.26
			
BMI (mean ± SE)	44 ± 2.7	35.4 ± 1.6	*p* = 0.01
			
Sex Ratio F:M	14:10 (58% F)	15:8 (68% F)	

### Urinary norepinephrine and fecal calprotectin

**Figure 2:**
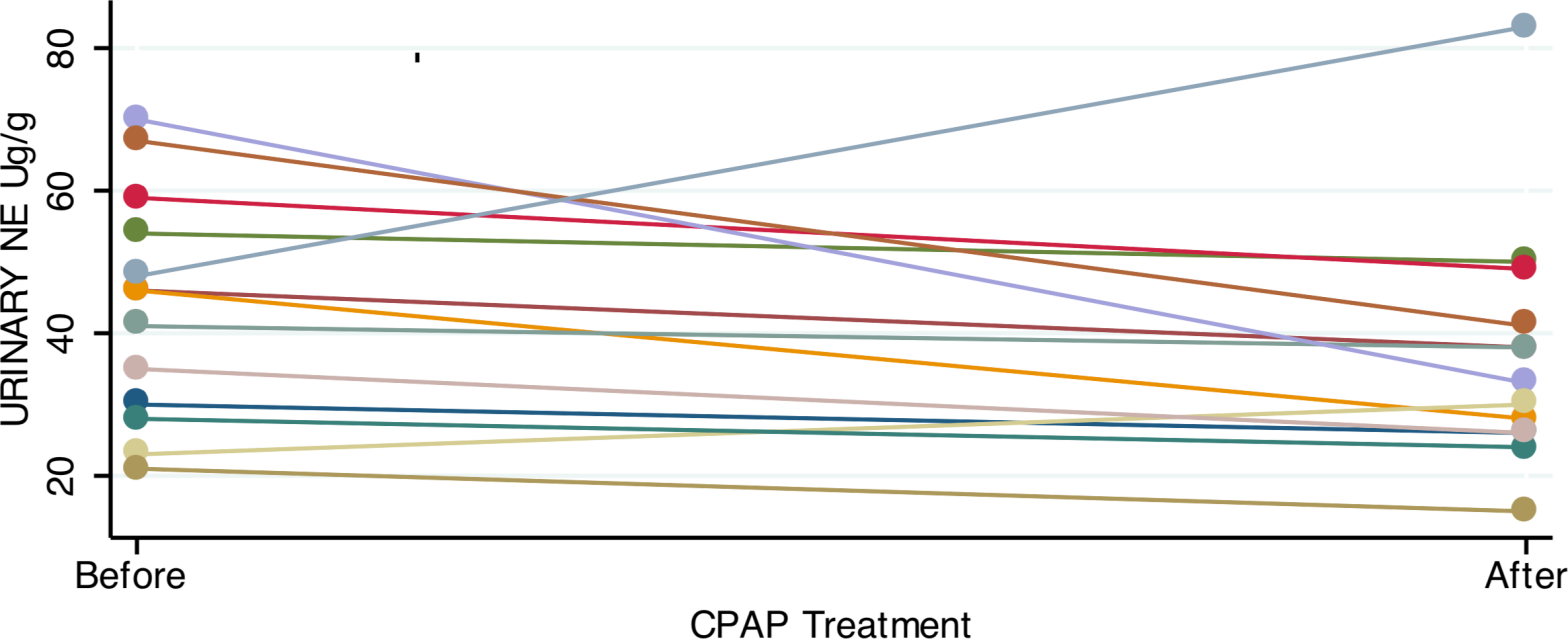
Urinary norepinephrine before and after CPAP treatment. Most OSA patients showed an absolute reduction in urinary norepinephrine after treatment with 4+ weeks of CPAP. However, the mean reduction in urine norepinephrine was not significant (paired *t*-test; *p* = 0.17).

Consistent with previous studies showing elevated catecholamines in sleep apnea, patients had significantly higher urinary norepinephrine relative to controls (Table 2). For patients with OSA prior to treatment, the 24-hour urinary excretion of norepinephrine, corrected for creatinine excretion, was 44.2 ± 3.54 µg/g (mean, SE). Of the 23 controls, 22 provided adequate urine for analysis. Controls had lower 24-hour urinary norepinephrine, corrected for creatinine: 29.5 ± 3 µg/g (mean, SE; *p* = 0.003, two tailed t test). In OSA patients who gave both 1st and 2nd samples, the mean difference in urine norepinephrine (6.7 ± 4.7 µg/g) was not significantly lower after CPAP treatment (Fig. 2).

**Table 2:** Twenty-four hour urine norepinephrine in obstructive sleep apnea patients and controls. Urine was collected at two time points. For OSA patients these corresponded to pretreatment and post-treatment with continuous positive airway pressure. Urine NE was significantly higher in patients with obstructive sleep apnea (pre-treatment) compared to controls. At the second time point, after CPAP treatment, patients were not significantly different from controls.

24 Hour urinary norepinephrine			
First collection	OSA (*n* = 24)	Control (*n* = 22^*^)	
	44.2 ± 3.54 µg/g (mean, SE)	29.5 ± 3.0 µg/g (mean, SE)	*t* test, *p* = 0.003
			
Second collection	OSA (*n* = 13)	Control (*n* = 18)	
	37 ± 4.7 µg/g	32 ± 3.2 µg/g	*t* test, *p* = 0.41
Treatment effect	OSA before CPAP (*n* = 13)	OSA after CPAP (*n* = 13)	
	43.7 ± 4.4 µg/g	37.2 ± 4.7 µg/g	paired *t* test, *p* = 0.17

**Notes.**

* One control subject did not give sufficient urine for analysis.

**Figure 3:**
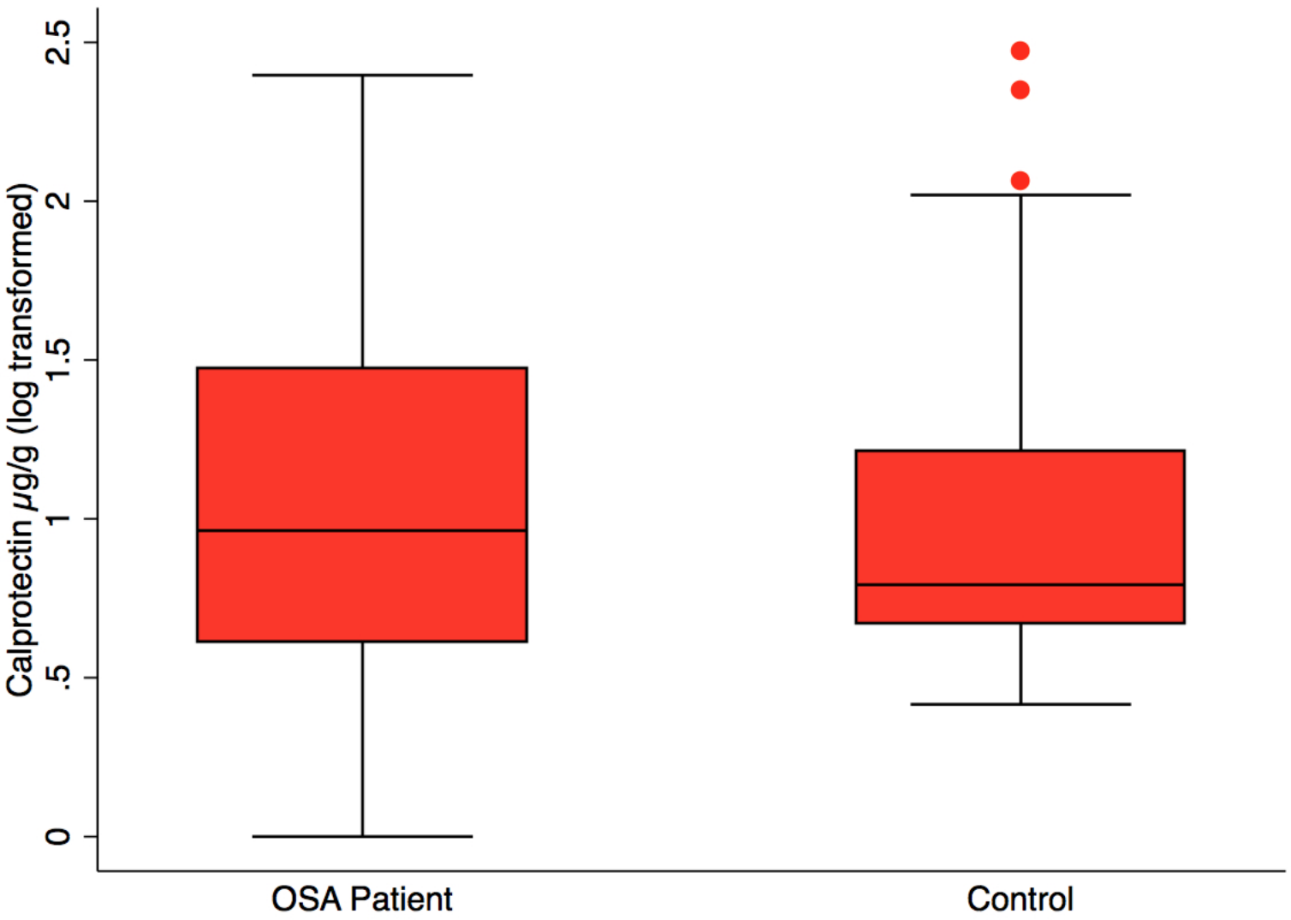
Fecal calprotectin by patient status. Box plot showing median, 25th and 75th percentile values for fecal calprotectin, a biomarker of intestinal inflammation. OSA patients did not differ from controls, *t* test, *p* = 0.37. OSA post-treatment did not differ from post-treatment, paired *t* test, *p* = 0.55, not shown.

Fecal calprotectin, measured to assess intestinal inflammation, was not significantly different in OSA (*n* = 24), 38.3 ±14.4 µg/g (mean, SE) *versus* controls (*n* = 23), 22.1 ± 10.2 µg/g, (mean, SE), *p* = 0.37 (Fig. 3). CPAP did not result in lower fecal calprotectin in the OSA patients who gave both 1st and 2nd collections (*p* = 0.55, paired *t*-test).

### Fecal enterobacteria

**Figure 4:**
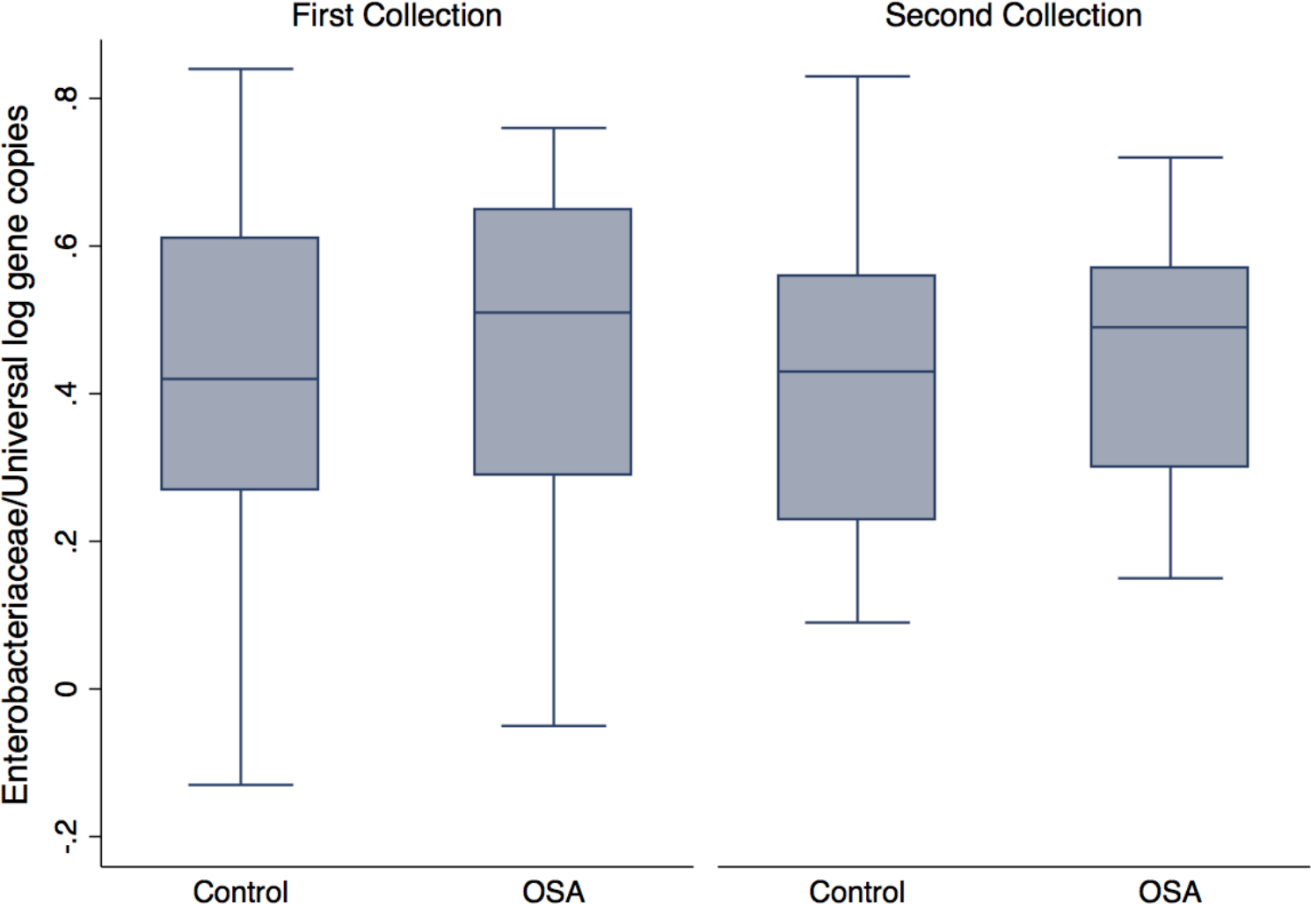
Enterobacteriaceae abundance by patient status. No differences were seen in Enterobacteriaceae abundance (log Enterobacteriaceae gene copies/log Universal Bacteria gene copies) in OSA patients compared to controls (1st sample collections, 0.45 ± 0.05 (*n* = 23) and 0.40 ± 0.05 (mean, SE, *n* = 23), respectively, *p* = 0.46, *t* test).

Quantitative PCR showed no differences in Enterobacteriaceae abundance in OSA patients compared to controls (1st sample collection only, *p* = 0.6, *t* test; Fig. 4). One OSA patient sample had unreliable fecal DNA qPCR results and was not included. Fecal Enterobacteriaceae abundance was calculated as the proportion of log Enterobacteriaceae gene copies/log Universal gene copies. Among OSA patients who gave both 1st and 2nd collections, CPAP treatment did not affect Enterobacteriaceae abundance (*p* = 0.4).

A linear mixed-effects model tested whether fecal Enterobacteriaceae abundance was influenced by the following variables: creatinine-corrected norepinephrine (NE), BMI, age, sex, and OSA patient status. The outcome variable was Enterobacteriaceae abundance, expressed as the number of Enterobacteriaceae gene copies, divided by the number of Universal gene copies in a sample. The mixed-effects generalized linear model allowed us to include all measurements for which we had complete data. These included OSA patient 1st collections (*n* = 23 excluding one individual with unreliable fecal DNA qPCR results) and OSA 2nd collections (*n* = 13) from OSA patients, as well as control subject 1st collections (*n* = 22, excluding one individual with inadequate urine for catecholamine analysis) and control 2nd collections (*n* = 18). A model that included all variables and interaction effects showed a positive association only for the interaction of OSA and NE (xtmixed procedure, *p* = 0.02). When stratified by patient status, a significant positive association between Enterobacteriaceae abundance and norepinephrine was found in the OSA patient group (*p* = 0.009) and not in controls (*p* = 0.2) (see Supplemental File for model details).

**Figure 5:**
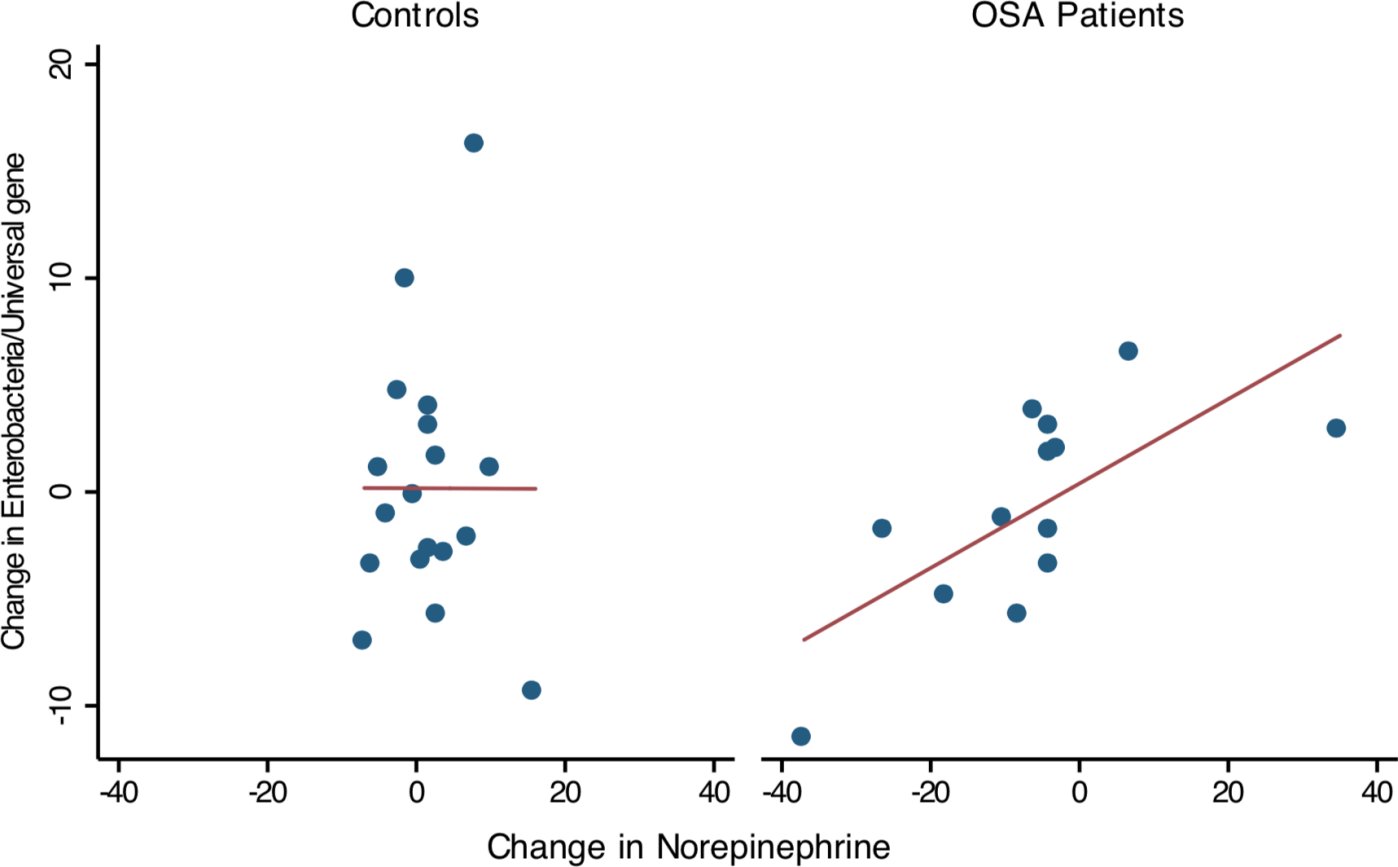
Change in Enterobacteriaceae abundance and urinary norepinephrine. Simple linear regression showed that within-subject change of Enterobacteriaceae abundance (Δ Enterobacteriaceae log gene copies/Universal Bacteria log gene copies) was positively associated to the corresponding change in urine norepinephrine (Δ NE *μ*g/g, corrected for creatinine excretion). Controls showed no significant relationship between Enterobacteriaceae abundance and urine norepinephrine.

Thirteen OSA patients provided 1st and 2nd collections (pre- and post-treatment), permitting comparison of within-subject changes in Enterobacteriaceae and urine norepinephrine. In OSA patients (*n* = 13), the change in Enterobacteriaceae abundance was strongly associated with the change in 24 h urine norepinephrine between 1st and 2nd collections (linear regression, *R*^2^ = 0.47, *p* = 0.01, Fig. 5). No such relationship was seen in controls who provided two specimen collections (*n* = 18, R^2^ = 0, Fig. 5).

### Beta and alpha diversity of the gut microbiota

**Figure 6:**
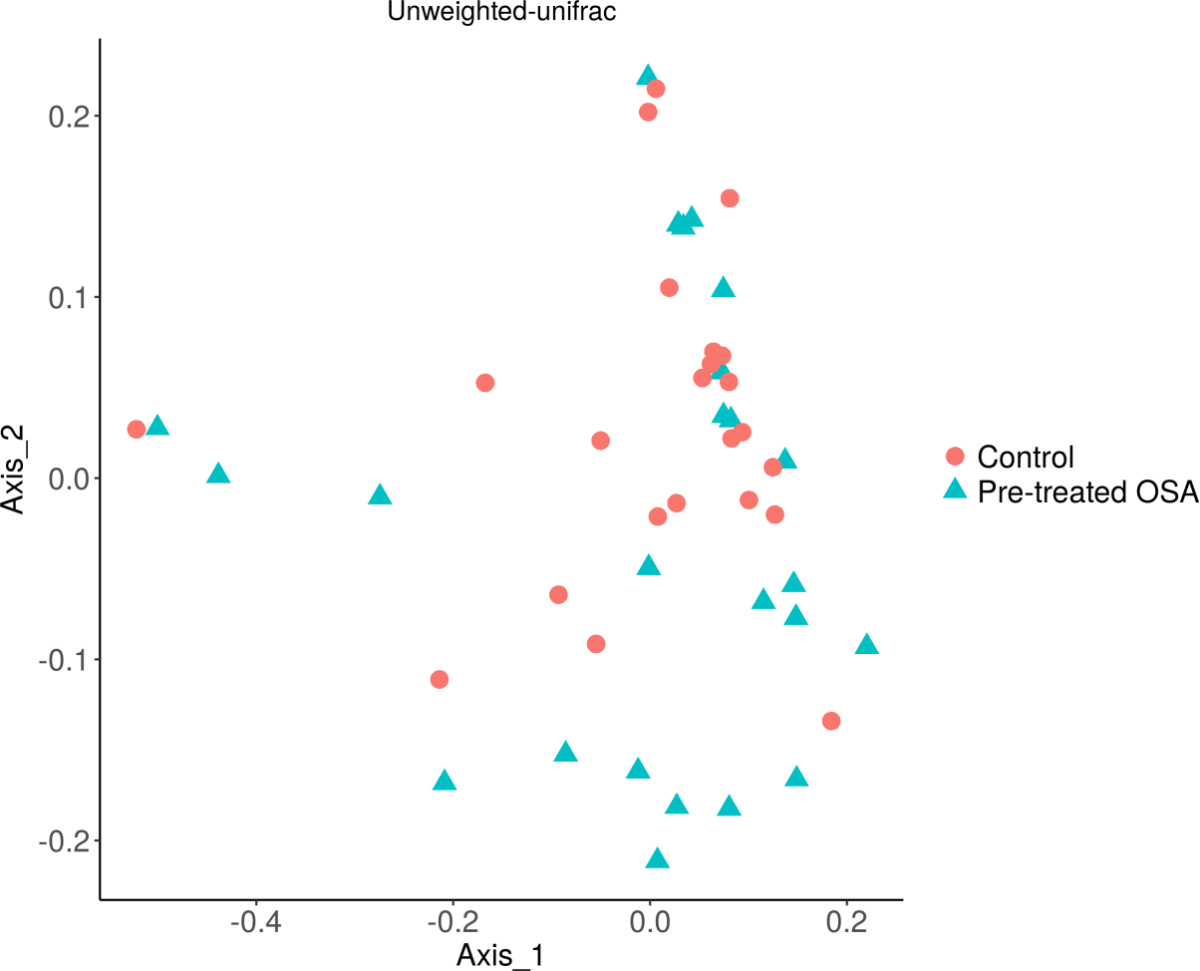
PCOA plot of community structure of the microbiome in OSA patients and controls. PCOA plot showing similar community structure of the fecal microbiota in OSA patients (first collection, *n* = 24) and controls (first fecal collection, *n* = 23), using unweighted unifrac (*p* = 0.56) and weighted unifrac (*p* = 0.4, not shown).

**Figure 7:**
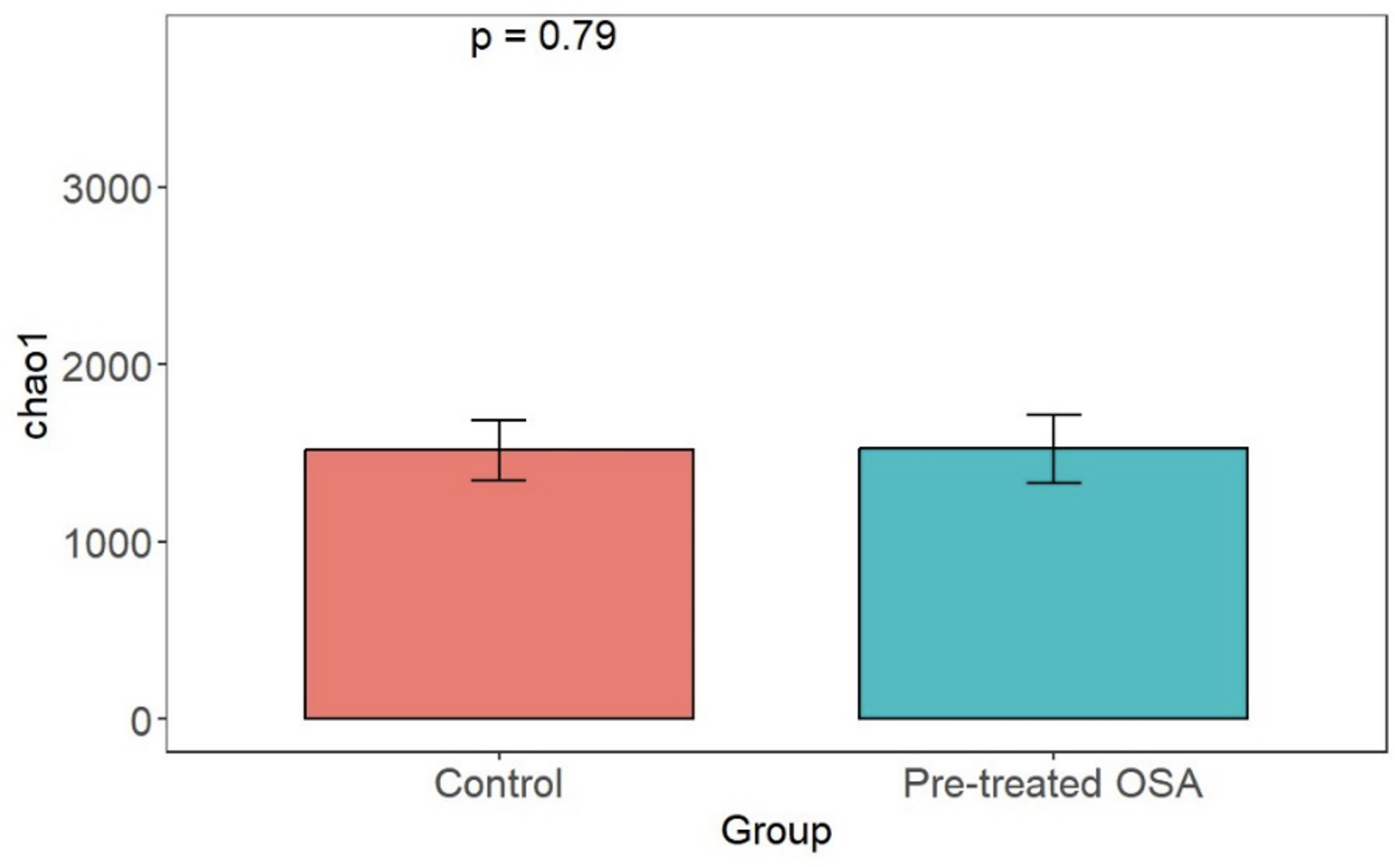
Chao1 alpha diversity. Overall microbiota richness in similar between patients and controls.

**Figure 8:**
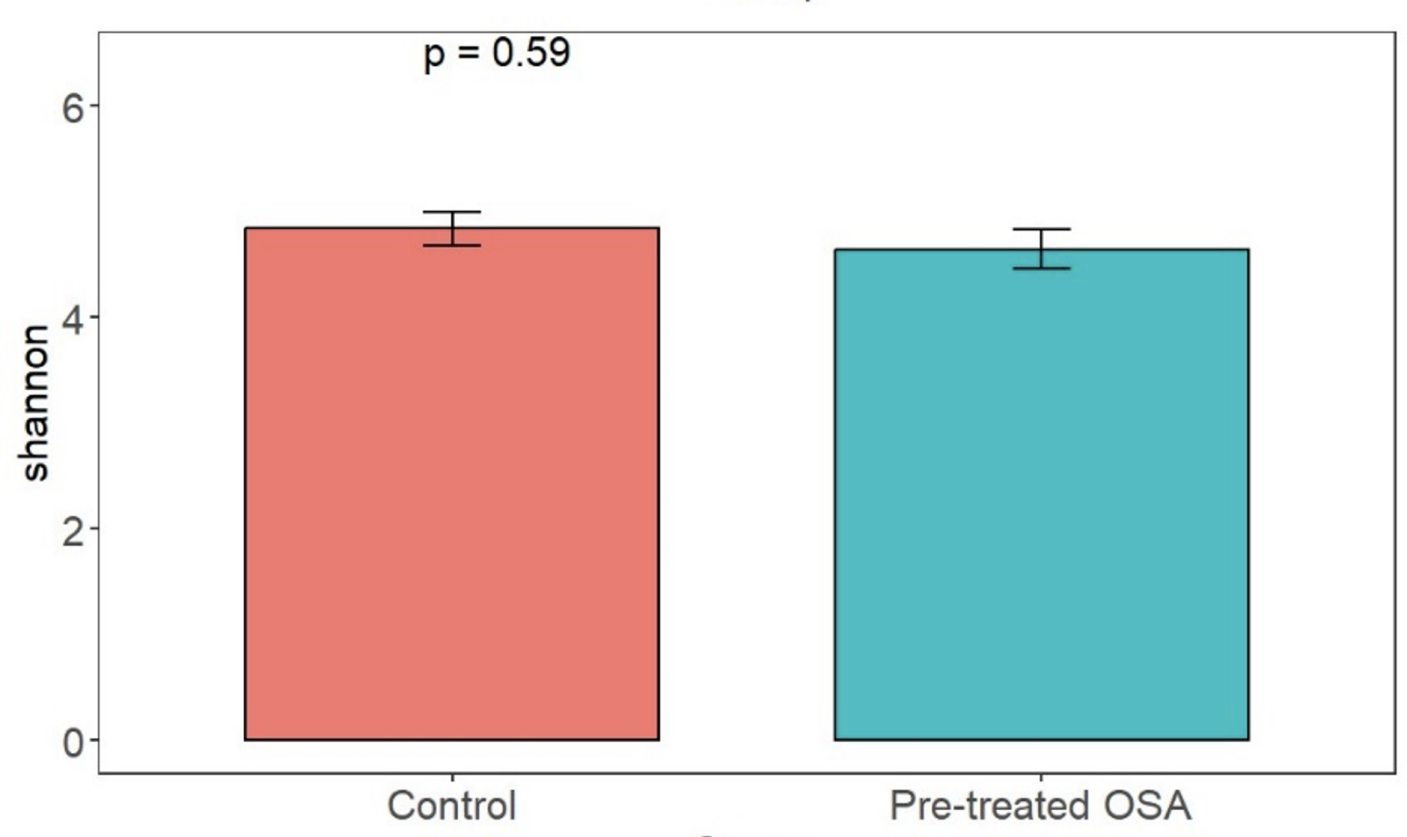
Shannon diversity index. Shannon index metrics were similar in patients and controls.

**Figure 9:**
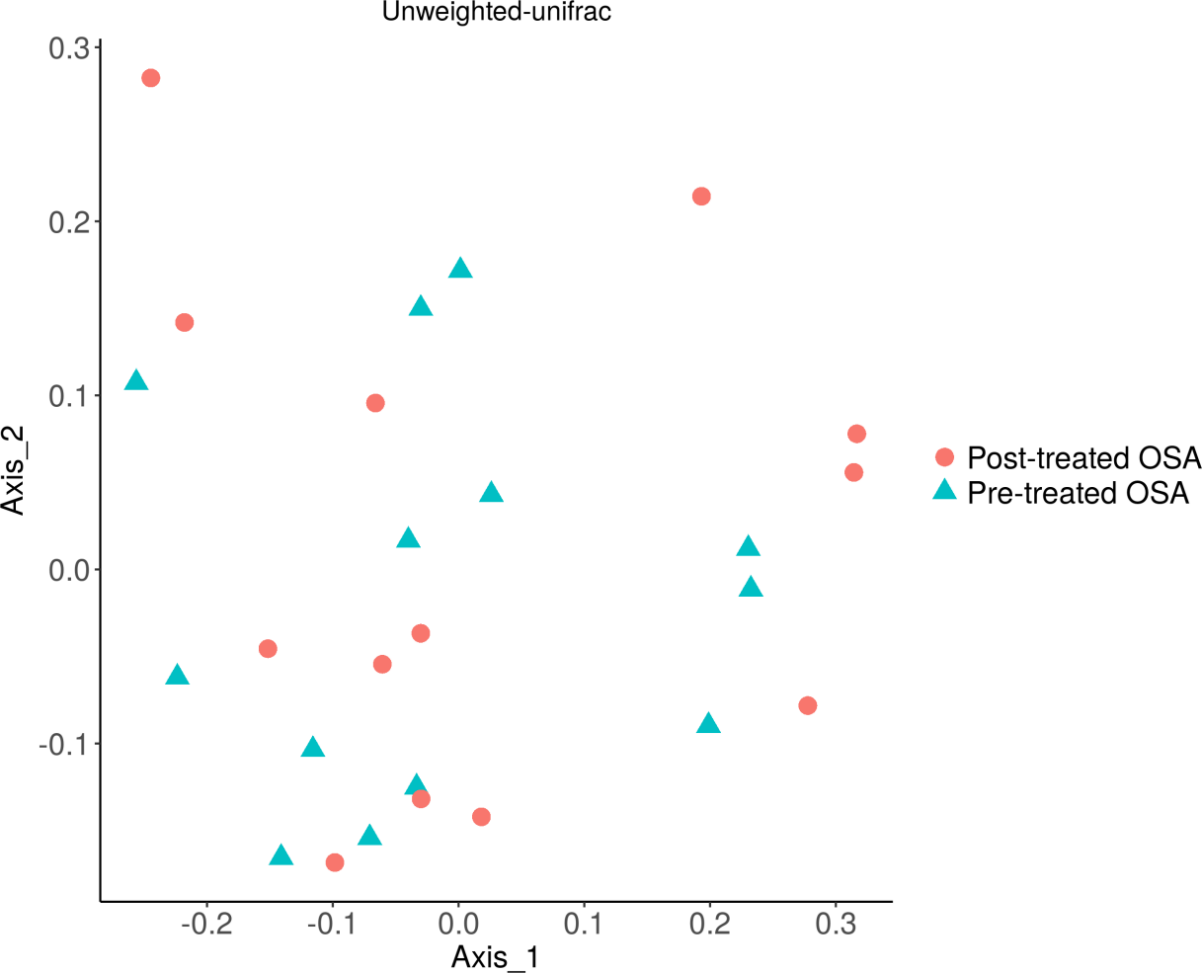
Unweighted UniFrac showing OSA microbiome community structure before and after treatment with CPAP. No difference was seen in the beta diversity of the fecal microbiome of OSA patients before and after treatment (unweighted unifrac; *p* = 0.9). Similarly, treatment status did not affect microbial abundance (weighted unifrac; *p* = 0.9, not shown).

There were 200 OTUs from 94 families identified and we applied 4000 for rarefaction and estimation of diversity. 16S Illumina sequencing revealed a similar community structure of the fecal microbiota in OSA patients compared to controls (1st collection only); Unweighted unifrac showed no significant difference in community structure, *p* = 0.56, Fig. 6). Weighted unifrac also indicated no significant difference between OSA and controls. Non-phylogenetic distance metrics similarly showed no differences between groups. Bray-Curtis dissimilarity index comparing OSA and controls was nonsignificant, *p* = 0.34 (Fig. S1). We used the alpha diversity metrics chao1 and Shannon index to assess gut microbiota richness and evenness in controls, patients, and patients treated with CPAP. These showed no differences between patients and controls (chao1 *p* = 0.79, Shannon index *p* = 0.59, Figs. 7, 8). No differences were seen using these indices in patients before and after CPAP treatment (chao1 *p* = 0.46, Shannon index *p* = 0.91; Fig. S2). In addition, no difference in the community structure of the fecal microbiome was seen between before and after CPAP treatment in OSA patients using unweighted unifrac (Fig. 9). Abundant genera in patients with OSA included members of Bacteroidetes phylum—Bacteroides, Paraprevotella, Parabacteroides, and Alistipes (Supplemental File); Proteobacteria were identified at lower abundance than would be predicted by the quantitative PCR results (above).

We contrasted the fecal microbiome of OSA patients who had high levels of norepinephrine, defined as greater than or equal to 40 µg/g urine (*n* = 11), and those with low levels of norepinephrine, less than 40 µg/g urine (*n* = 13). No difference in the microbiome community structure was observed between OSA patients with high and low urine norepinephrine levels (unweighted unifrac, *p* = 0.79; weighted unifrac, *p* = 0.34).

## Discussion

Increased production of stress catecholamines, particularly norepinephrine, has been observed in patients with obstructive sleep apnea and other sleep disorders (Elmasry et al., 2002; Phillips et al., 2007). Previous experimental work with lab animals showed that exposure to stress catecholamines can influence the abundance and invasiveness of gram-negative bacteria, including members of Enterobacteriaceae, *Desulfovibrio, and Fusobacterium* (Belay & Sonnenfeld, 2002; Coffman et al., 2022; Zhang et al., 2024). Significant cross talk exists between the gut microbiota and the host nervous system, and Lyte, Vulchanova & Brown (2011) have proposed that bacteria in the gut mucosa and lumen are influenced by norepinephrine and other neurochemicals produced by the host. This pilot study tested whether elevated norepinephrine in obstructive sleep apnea was associated altered gut microbiota, and whether treatment with continuous positive airway pressure might affect norepinephrine levels and the gut microbiota.

In animal studies of sleep deprivation, Enterobacteriaceae were the bacteria most often associated with intestinal bacterial overgrowth (Everson & Toth, 2000). Enterobacteriaceae and the phylum that includes them, Proteobacteria, were higher in OSA in some studies (Ko et al., 2019; Bikov et al., 2022; Chuang et al., 2024) but not in others, including the largest study to date (Baldanzi et al., 2023). In our study, OSA patients did not show significant differences in alpha or beta diversity overall. In addition, Enterobacteriaceae abundance in stool samples was not significantly increased overall in OSA patients or changed with CPAP treatment. OSA did not affect intestinal inflammation, as measured by fecal calprotectin. Nevertheless, we found that within-individual changes in the abundance of Enterobacteriaceae measured by qPCR among OSA patients were significantly associated with urine norepinephrine measurements taken at two time points, before and after treatment with CPAP. These findings should be viewed with caution considering the nonsignificant differences in beta and alpha diversity between groups in this study.

A longitudinal association between norepinephrine and Enterobacteriaceae was seen in subjects with obstructive sleep apnea but not in controls, suggesting that bacteria may be sensitive to norepinephrine only when patients have a chronic illness. However, it is possible that some other variable, such as apnea or hypoxia, caused the observed relationship. Arguing against this view, the apnea hypopnea index in OSA patients was not associated with Enterobacteriaceae abundance (*R*^2^ 0.05, *p* = 0.28). Another potential confounder, BMI, was not significantly associated with fecal Enterobacteriaceae in the mixed effects model that we performed. Instead, norepinephrine was significantly associated with Enterobacteriaceae in this study, supporting the view that catecholamines might have an influence on the microbiota in obstructive sleep apnea patients.

Previously published cross-sectional studies of the microbiota in OSA have shown varying results. Ko et al. (2019) reported that OSA was associated with an increase in Proteobacteria, among other taxa, but not with microbial richness. Valentini et al. (2020) showed that OSA in children was significantly associated with decreased alpha diversity and higher abundance of Desulfovibrionaceae and Proteobacteria in children with lower oxygen saturation. Li et al. (2023) showed a potential role for gram-negative *Fusobacterium* in patients with moderate and severe obstructive sleep apnea. A cross sectional study of 4045 patients with OSA reported higher Lachnospiraceae and *Fusobacterium* in patients with obstructive sleep apnea, though the association with *Fusobacterium* disappeared after correction for BMI (Baldanzi et al., 2023). A recent study stratified subjects by severity of OSA and found higher Lachnospiraceae, decreased *Faecalibacterium* and decreased alpha diversity among patients with severe OSA (Wang et al., 2024). The absence of a consistent gram-negative microbial fingerprint of OSA, along with the null findings in our study, raise some question about whether these taxa are enriched in OSA, as would be expected if increased exposure to catecholamines promotes their growth.

A strength of our study is the inclusion of repeated measures before and after CPAP treatment and a control group also measured longitudinally. Unlike our research, Baldanzi et al. (2023) did not include longitudinal measures nor the effect of treatment. By contrast, Bock et al. (2024) reported the results of eight patients with OSA before and after treatment with positive airway pressure. In line with our results, they found no significant effect of CPAP treatment on alpha or beta diversity (Bock et al., 2024). Our study involved slightly more OSA patients treated with CPAP than in Bock et al. (2024). However, inter-subject variability was substantial, and our study may have been underpowered to show differences in alpha and beta diversity.

One limitation of this study is that controls did not undergo polysomnography. Because sleep disorders were excluded in the control group only by self-report it is possible that some in the control group may have undiagnosed obstructive sleep apnea even though controls reported no sleep symptoms. In addition, we did not measure apnea or oxygenation variables after initiation of CPAP treatment. This limited our ability to test the effect of apnea and hypopnea on microbiota and other outcomes. Uncertainty exists around the duration of CPAP needed to change the microbiome. We chose a relatively short interval (>4 weeks) because CPAP results in systemic changes to norepinephrine in a week (Phillips et al., 2007) and other interventions, *e.g.*, diet, result in a change in the microbiota in as little as 24 h (Leeming et al., 2019). Whether CPAP can alter the microbiota remains uncertain. A recent study involving 8 patients had null results after two months of PAP (Bock et al., 2024), in line with our findings. An additional limitation is that we did not measure sympathetic activation directly. Norepinephrine in urine is an indirect marker of sympathetic nerve traffic. The present study’s qPCR results suggested higher abundance of Enterobacteriaceae than did sequencing results (4% of reads in patients, 4.8% of reads in controls); these differences may reflect limitations of those techniques (Louca, Doebeli & Parfrey, 2018). Given these limitations, the main positive finding of this study—an association between urinary norepinephrine and fecal Enterobacteriaceae in obstructive sleep apnea—should be viewed as a hypothesis-generating result. A larger prospective study will be needed to probe further whether norepinephrine has a causal relationship with intestinal Enterobacteriaceae or other members of the gut microbiota.

This research area has potential implications for other sleep conditions in addition to sleep apnea. For instance, short-term sleep deprivation has been reported to increase plasma norepinephrine levels (Irwin et al., 1999) and causes changes in metabolism that lead to weight gain (Markwald et al., 2013). The interplay between stress hormones and gut microbiota remains a largely unstudied topic in sleep disorders and circadian disruption.

## Conclusions

This study attempted to translate the robust preclinical literature showing potentially harmful effects of catecholamines on gram negative pathogens to obstructive sleep apnea, a common clinical condition that is characterized by elevated catecholamines. Although we found higher urinary norepinephrine among patients with obstructive sleep apnea, patients did not show significant changes of the community structure of the gut microbiota compared to controls. Treatment of obstructive sleep apnea with continuous positive airway pressure also was not associated with significant changes in the composition of the gut microbiota. However, within-subject longitudinal measurements showed a significant association of fecal Enterobacteriaceae measured by quantitative PCR with urinary norepinephrine in OSA patients. This result will require confirmation by future studies.

##  Supplemental Information

10.7717/peerj.19203/supp-1 Supplemental Information 1 Representation of microbial taxa in OSA patients 16S sequencing suggests that Firmicutes and Bacteroidetes are the dominant phyla in patients with OSA. Bacteroidetes makes up 17.6% of all reads. Proteobacteria accounts for 4.1% of total reads.

10.7717/peerj.19203/supp-2 Supplemental Information 2 Microbial taxa in controls 16S sequencing suggests that Firmicutes and Bacteroidetes are the dominant phyla in control subjects. Bacteroidetes was 25.5% of all reads and Proteobacteria occurred at 4.83%.

10.7717/peerj.19203/supp-3 Supplemental Information 3 Primers used in this study PCR amplification for each target was performed using the primers shown above.

10.7717/peerj.19203/supp-4 Supplemental Information 4 Bray Curtis non-phylogenetic beta diversity metric Bray Curtis showed no difference in beta diversity between OSA patients and controls.

10.7717/peerj.19203/supp-5 Supplemental Information 5 Shannon index in obstructive sleep apnea patients before and after treatment with CPAP No difference in microbiota richness was observed.

10.7717/peerj.19203/supp-6 Supplemental Information 6 Chao1 alpha diversity metric in obstructive sleep apnea patients with and without CPAP treatment Chao1 richness was similar in treated and untreated patients with obstructive sleep apnea.

10.7717/peerj.19203/supp-7 Supplemental Information 7 Calprotectin assay results Calprotectin isolated from human fecal samples in subjects with obstructive sleep apnea and controls.

10.7717/peerj.19203/supp-8 Supplemental Information 8 Subject demographics, 24 hour catecholamines, polysomnogram results

10.7717/peerj.19203/supp-9 Supplemental Information 9 NCBI Sequence Read Archive Information

10.7717/peerj.19203/supp-10 Supplemental Information 10 Mixed effects linear model Mixed effects linear model stratified by patient status (o = control, 1 = OSA patient) including independent variables creatinine-corrected norepinephrine (neugg), body mass index (bmi), and age in years (age). The dependent variable is Enterobacteria, expressed as gene copies of Enterobacteriaceae divided by universal bacteria gene copies (unienteroratio). (A) For controls, the model results in a non-significant effect of norepinephrine, bmi, and age on enterobacteria. (B) Patients with OSA show a significant effect of norepinephrine on Enterobacteria (*p* = 0.01). Bmi and age remain nonsignificant.

## Additional Information and Declarations

### Data Availability

The following information was supplied regarding data availability: The sequences for this project are available at the NCBI SRA: SRP077632.
