# Supplementary material for: Catecholamine exposure and the gut microbiota in obstructive sleep apnea
Source: PeerJ. 2025 Apr 14;13:e19203. doi: 10.7717/peerj.19203 (PMC12005174; doi:10.7717/peerj.19203)
Supplement: Supplemental Information 1 — 16S sequencing suggests that Firmicutes and Bacteroidetes are the dominant phyla in patients with OSA. Bacteroidetes makes up 17.6% of all reads. Proteobacteria accounts for 4.1% of total reads. [file peerj-13-19203-s001.docx]

| taxon | total | total OSA patient reads | percentage of all reads |
| --- | --- | --- | --- |
| Bacteria | 5389 | 10321 |  |
| Firmicutes | 3205 | 7222 | 69.97% |
| Bacteroidetes | 1394 | 1814 | 17.58% |
| unclassified | 370 | 429 | 4.16% |
| Proteobacteria | 180 | 420 | 4.07% |
| Actinobacteria | 171 | 357 | 3.46% |
| Fusobacteria | 11 | 15 | 0.15% |
| Lentisphaerae | 3 | 8 | 0.08% |
| Synergistetes | 13 | 6 | 0.06% |
| Gemmatimonadetes | 1 | 5 | 0.05% |
| Spirochaetes | 3 | 3 | 0.03% |
| Acidobacteria | 1 | 2 | 0.02% |
| TM7 | 1 | 1 | 0.01% |
| Deinococci | 1 | 1 | 0.01% |
| Sphingobacteria | 2 | 0 | 0 |
| Planctomycetes | 1 | 0 | 0 |
