# Supplementary material for: Catecholamine exposure and the gut microbiota in obstructive sleep apnea
Source: PeerJ. 2025 Apr 14;13:e19203. doi: 10.7717/peerj.19203 (PMC12005174; doi:10.7717/peerj.19203)
Supplement: Supplemental Information 2 — 16S sequencing suggests that Firmicutes and Bacteroidetes are the dominant phyla in control subjects. Bacteroidetes was 25.5% of all reads and Proteobacteria occurred at 4.83%. [file peerj-13-19203-s002.docx]

| taxon | total | Total Control reads | Percentage of all reads |
| --- | --- | --- | --- |
| Bacteria | 5389 | 11780 |  |
| Firmicutes | 3205 | 7731 | 74.91 |
| Bacteroidetes | 1394 | 2634 | 25.52 |
| unclassified | 370 | 448 | 4.34 |
| Proteobacteria | 180 | 499 | 4.83 |
| Actinobacteria | 171 | 355 | 3.44 |
| Fusobacteria | 11 | 13 | 0.13 |
| Lentisphaerae | 3 | 8 | 0.08 |
| Synergistetes | 13 | 14 | 0.14 |
| Gemmatimonadetes | 1 | 5 | 0.05 |
| Spirochaetes | 3 | 0 | 0.00 |
| Acidobacteria | 1 | 0 | 0.00 |
| TM7 | 1 | 0 | 0.00 |
| Deinococci | 1 | 0 | 0.00 |
| Sphingobacteria | 2 | 3 | 0.03 |
| Planctomycetes | 1 | 2 | 0.02 |
