# Supplementary material for: Catecholamine exposure and the gut microbiota in obstructive sleep apnea
Source: PeerJ. 2025 Apr 14;13:e19203. doi: 10.7717/peerj.19203 (PMC12005174; doi:10.7717/peerj.19203)
Supplement: Supplemental Information 3 — PCR amplification for each target was performed using the primers shown above. [file peerj-13-19203-s003.docx]

| Table | Primer | Sequence (5′→3′) | References |
| --- | --- | --- | --- |
| Total bacteria 16S rRNA gene Universal | F1048  (forward) | GTG (GC)TG CA(CT) GG(CT) TGT CGT CA | (Carroll et al., 2010) |
|  | R1119  (reverse) | ACG TC(AG) TCC (AC)CA CCT TCC TC | (Carroll et al., 2010) |
| Enterobacteria, Escherichia coli (Enterobacteriaceae) | Eco1457  (forward) | CATTGACGTTACCCGCAGAAGAAGC | (Bartosch et al., 2004) |
|  | Eco1652 (reverse) | CTCTACGAGACTCAAGCTTGC | (Bartosch et al., 2004) |
