# Supplementary figures and images for: Catecholamine exposure and the gut microbiota in obstructive sleep apnea
Source: PeerJ. 2025 Apr 14;13:e19203. doi: 10.7717/peerj.19203 (PMC12005174; doi:10.7717/peerj.19203)

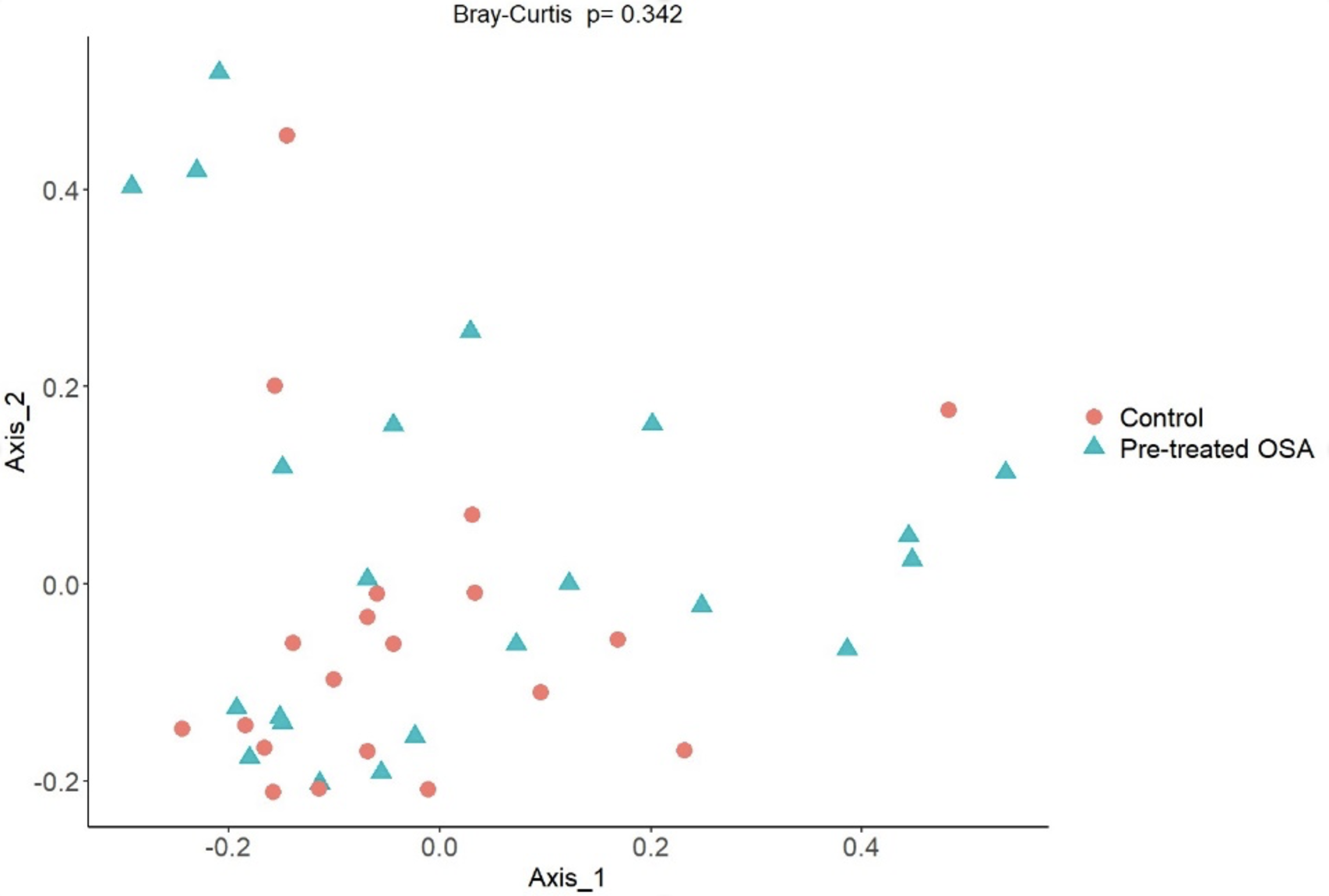

Supplement: Supplemental Information 4 — Bray Curtis showed no difference in beta diversity between OSA patients and controls. [file peerj-13-19203-s004.png]

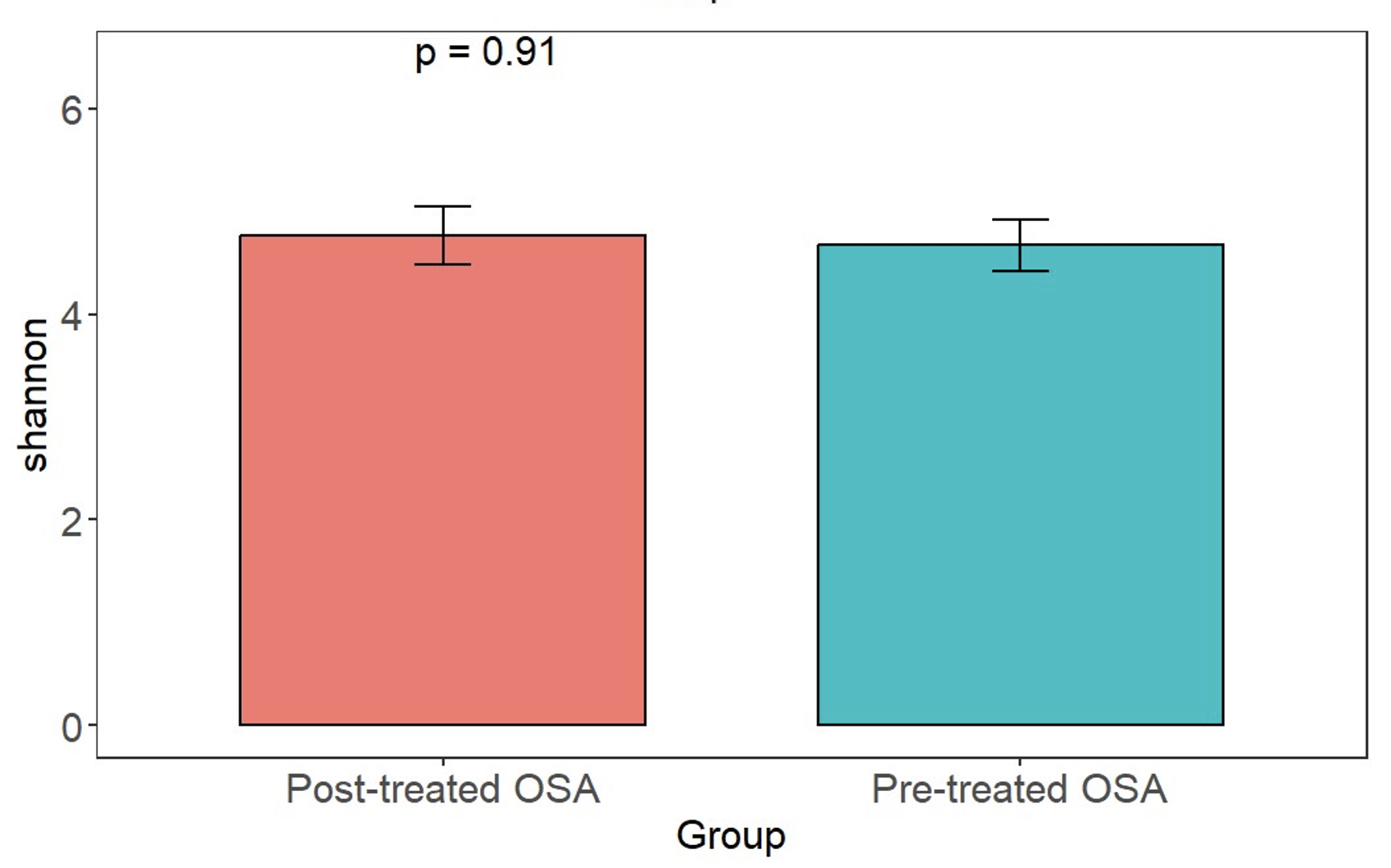

Supplement: Supplemental Information 5 — No difference in microbiota richness was observed. [file peerj-13-19203-s005.png]

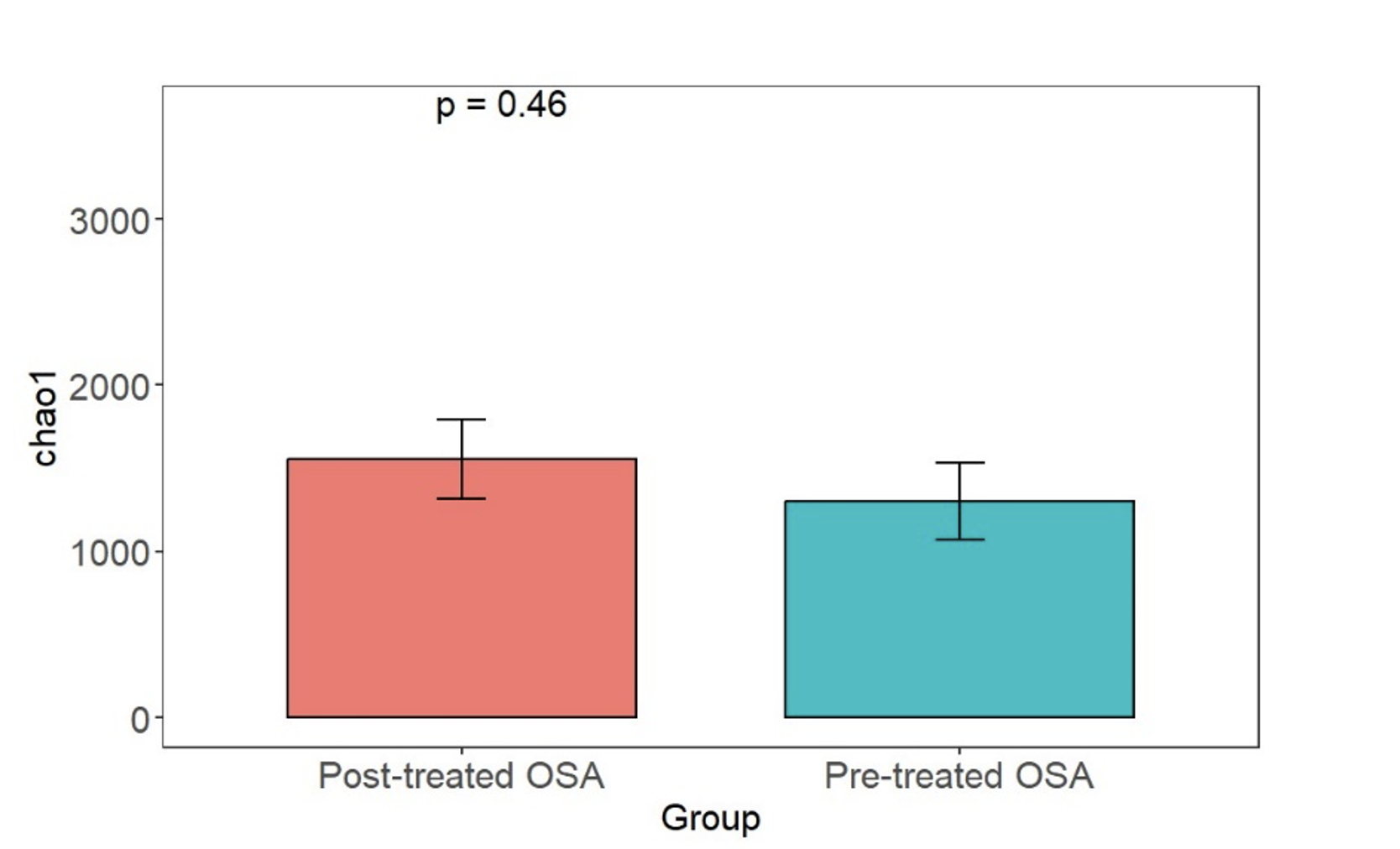

Supplement: Supplemental Information 6 — Chao1 richness was similar in treated and untreated patients with obstructive sleep apnea. [file peerj-13-19203-s006.png]

(A)


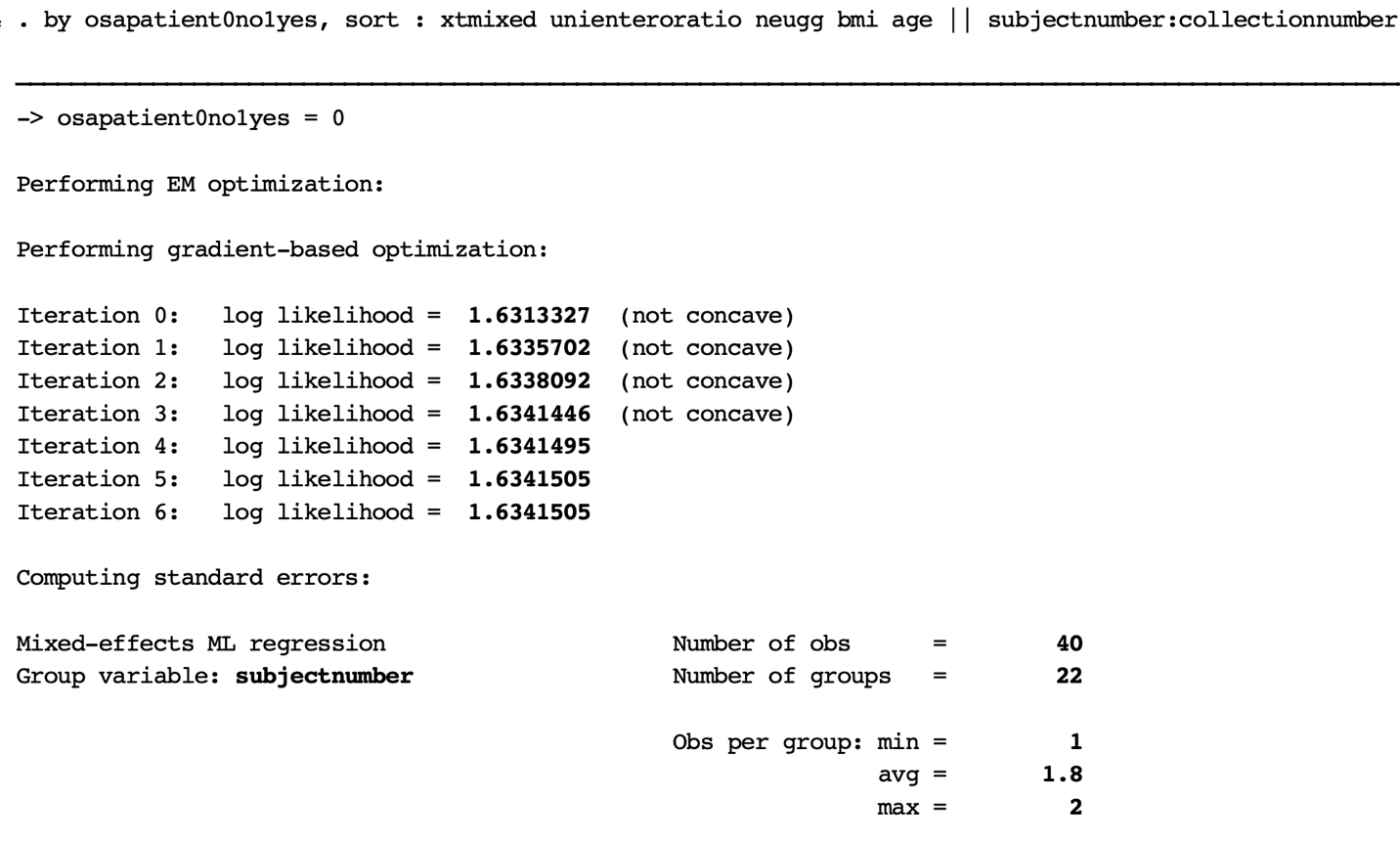


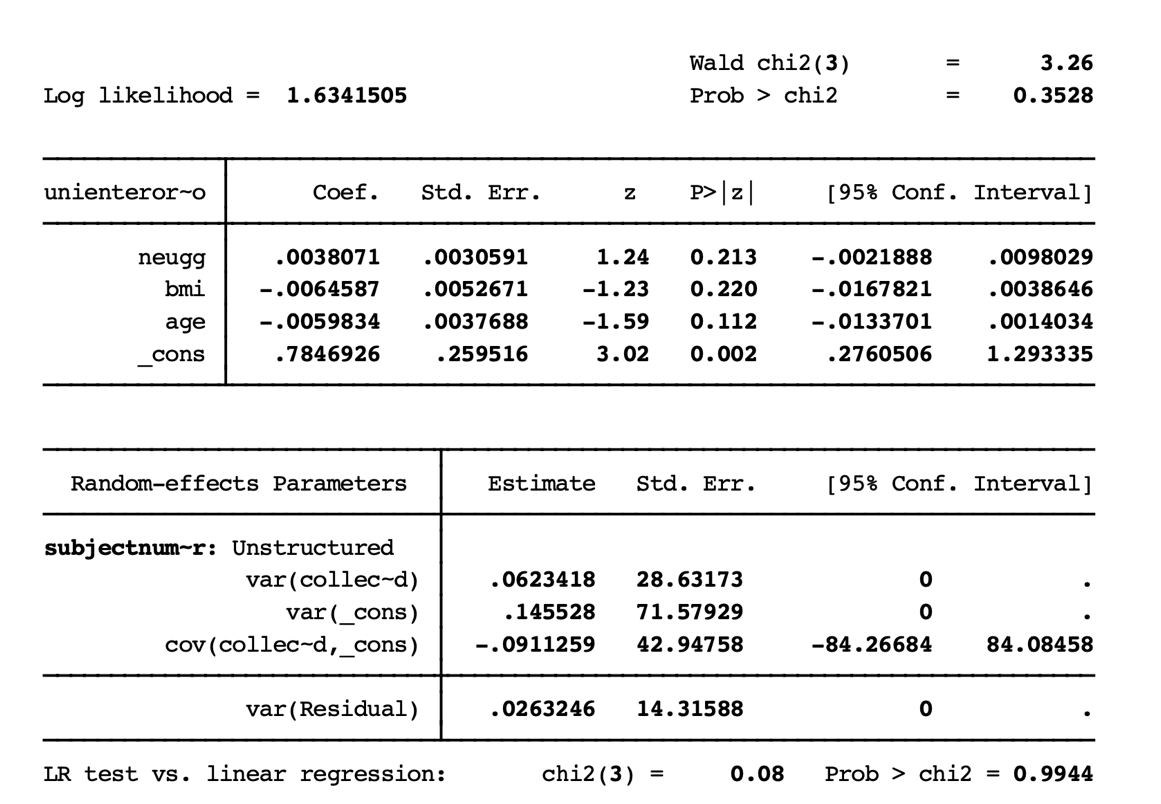


(B)


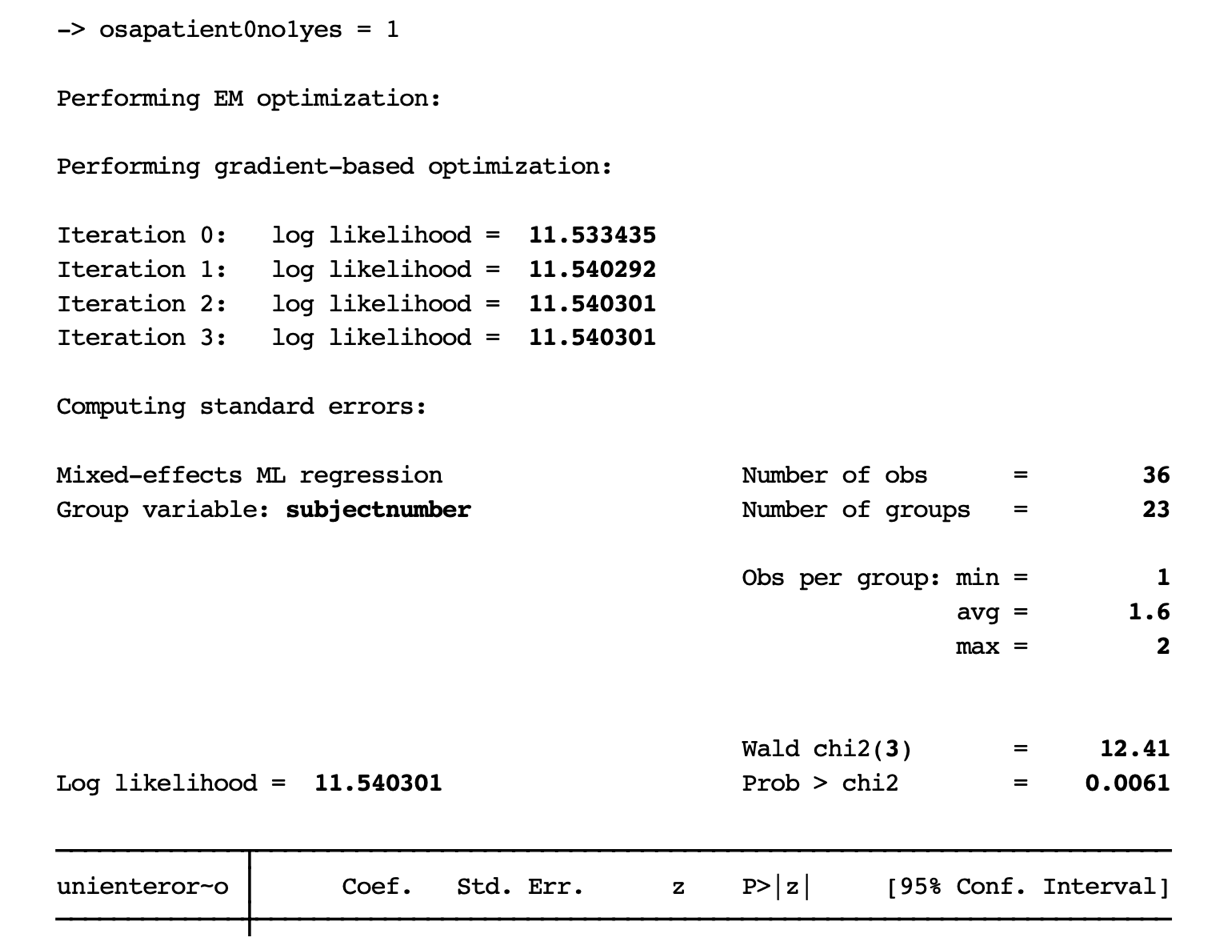


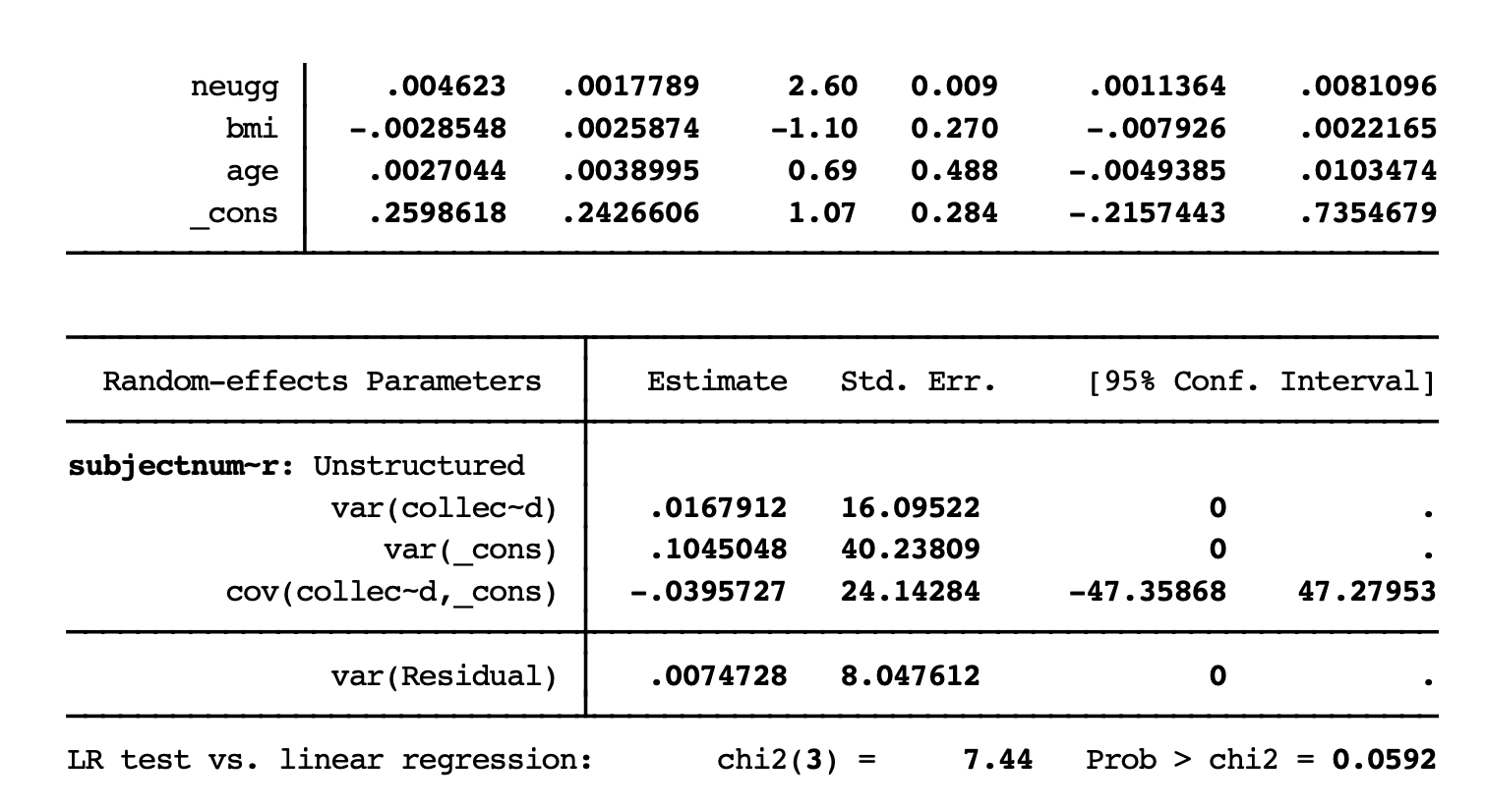

Supplement: Supplemental Information 10 — Mixed effects linear model stratified by patient status (o = control, 1 = OSA patient) including independent variables creatinine-corrected norepinephrine (neugg), body mass index (bmi), and age in years (age). The dependent variable is Enterobacteria, expressed as gene copies of Enterobacteriaceae divided by universal bacteria gene copies (unienteroratio). (A) For controls, the model results in a non-significant effect of norepinephrine, bmi, and age on enterobacteria. (B) Patients with OSA show a significant effect of norepinephrine on Enterobacteria (p = 0.01). Bmi and age remain nonsignificant. [file peerj-13-19203-s010.docx]
