# Supplementary material for: Catecholamine exposure and the gut microbiota in obstructive sleep apnea
Source: PeerJ. 2025 Apr 14;13:e19203. doi: 10.7717/peerj.19203 (PMC12005174; doi:10.7717/peerj.19203)
Supplement: Supplemental Information 9 [file peerj-13-19203-s009.pdf]

Submission: SUB1657530

Submission Id	Submitter	Updated	State	Status	Comments
Los Alamos National Laboratory : SUB1657530	Shannon L. Johnson	2016-06-30 10:57	completed	238	• SRP077632 : PRJNA323360<br>• 79 samples<br>• 79 experiments<br>• 79 runs

Files

Type	Alias	Accession	Uploaded	Links	Files	Released
STUDY	PRJNA323360	SRP077632	76 m	ok	done	
SAMPLE	HMC_119	SRS1531682	74 m	ok	done	
EXPERIMENT New Run	HMC_119	SRX1888293	77 m	ok	done	
RUN	HMC_100_000000000-A8DJ9_ACACGTAAGCCT_L001_R1_001.fastq.gz	SRR3732477	76 m	ok	done	2017-07-03 00:00:00
SAMPLE	HMC_120	SRS1531683	74 m	ok	done	
EXPERIMENT New Run	HMC_120	SRX1888294	77 m	ok	done	
RUN	HMC_101_000000000-A8DJ9_GAGTGGTAGAGA_L001_R2_001.fastq.gz	SRR3732478	76 m	ok	done	2017-07-03 00:00:00
SAMPLE	HMC_102.1	SRS1531684	74 m	ok	done	
EXPERIMENT New Run	HMC_102.1	SRX1888295	77 m	ok	done	
RUN	HMC_110_000000000-A8DJ9_GACGGAACCCAT_L001_R1_001.fastq.gz	SRR3732479	76 m	ok	done	2017-07-03 00:00:00
SAMPLE	HMC_103.1	SRS1531685	74 m	ok	done	
EXPERIMENT New Run	HMC_103.1	SRX1888296	77 m	ok	done	
RUN	HMC_111_000000000-A8DJ9_CAAGCATGCCTA_L001_R2_001.fastq.gz	SRR3732480	76 m	ok	done	2017-07-03 00:00:00
SAMPLE	HMC_104.1	SRS1531686	74 m	ok	done	
EXPERIMENT New Run	HMC_104.1	SRX1888297	77 m	ok	done	
RUN	HMC_112_000000000-A8DJ9_CCTGAACTAGTT_L001_R2_001.fastq.gz	SRR3732481	76 m	ok	done	2017-07-03 00:00:00
SAMPLE	HMC_106.1	SRS1531687	74 m	ok	done	
EXPERIMENT New Run	HMC_106.1	SRX1888298	77 m	ok	done	
RUN	HMC_113_000000000-A8DJ9_CTTCGGCAGAAT_L001_R2_001.fastq.gz	SRR3732482	76 m	ok	done	2017-07-03 00:00:00
SAMPLE	HMC_107.1	SRS1531688	74 m	ok	done	
EXPERIMENT New Run	HMC_107.1	SRX1888299	77 m	ok	done	
RUN	HMC_114_000000000-A8DJ9_ACGGGACATGCT_L001_R2_001.fastq.gz	SRR3732483	76 m	ok	done	2017-07-03 00:00:00
SAMPLE	HMC_108.1	SRS1531689	74 m	ok	done	

Type	Alias	Accession	Uploaded	Links	Files	Released
EXPERIMENT<br>New Run	HMC_108.1	SRX1888300	77 m	ok	done	
RUN	HMC_115_000000000-A8DJ9_GTCATATCGTAC_L001_R1_001.fastq.gz	SRR3732484	76 m	ok	done	2017-07-03 00:00:00
SAMPLE	HMC_110.1	SRS1531690	74 m	ok	done	
EXPERIMENT<br>New Run	HMC_110.1	SRX1888301	77 m	ok	done	
RUN	HMC_116_000000000-A8DJ9_GGAAACCACCAC_L001_R2_001.fastq.gz	SRR3732485	76 m	ok	done	2017-07-03 00:00:00
SAMPLE	HMC_112.1	SRS1531691	74 m	ok	done	
EXPERIMENT<br>New Run	HMC_112.1	SRX1888302	77 m	ok	done	
RUN	HMC_117_000000000-A8DJ9_TTGCGCATACTA_L001_R1_001.fastq.gz	SRR3732486	76 m	ok	done	2017-07-03 00:00:00
SAMPLE	HMC_114.1	SRS1531692	74 m	ok	done	
EXPERIMENT<br>New Run	HMC_114.1	SRX1888303	77 m	ok	done	
RUN	HMC_118_000000000-A8DJ9_ACATTCAGCGCA_L001_R1_001.fastq.gz	SRR3732487	76 m	ok	done	2017-07-03 00:00:00
SAMPLE	HMC_116.1	SRS1531693	74 m	ok	done	
EXPERIMENT<br>New Run	HMC_116.1	SRX1888304	77 m	ok	done	
RUN	HMC_119_000000000-A8DJ9_ACTGACAGCCAT_L001_R1_001.fastq.gz	SRR3732488	76 m	ok	done	2017-07-03 00:00:00
SAMPLE	HMC_121	SRS1531694	74 m	ok	done	
EXPERIMENT<br>New Run	HMC_121	SRX1888305	77 m	ok	done	
RUN	HMC_102_000000000-A8DJ9_GAAGTTGGAAGT_L001_R1_001.fastq.gz	SRR3732489	76 m	ok	done	2017-07-03 00:00:00
SAMPLE	HMC_119.1	SRS1531695	74 m	ok	done	
EXPERIMENT<br>New Run	HMC_119.1	SRX1888306	76 m	ok	done	
RUN	HMC_120_000000000-A8DJ9_CGAGAAGAGAAC_L001_R2_001.fastq.gz	SRR3732490	76 m	ok	done	2017-07-03 00:00:00
SAMPLE	HMC_120.1	SRS1531696	74 m	ok	done	
EXPERIMENT<br>New Run	HMC_120.1	SRX1888307	76 m	ok	done	
RUN	HMC_121_000000000-A8DJ9_AGGCATCTTACG_L001_R1_001.fastq.gz	SRR3732491	76 m	ok	done	2017-07-03 00:00:00
SAMPLE	HMC_121.1	SRS1531697	74 m	ok	done	
EXPERIMENT<br>New Run	HMC_121.1	SRX1888308	76 m	ok	done	
RUN	HMC_122_000000000-A8DJ9_CAGCTAGAACGC_L001_R1_001.fastq.gz	SRR3732492	76 m	ok	done	2017-07-03 00:00:00
SAMPLE	HMC_122.1	SRS1531698	74 m	ok	done	
EXPERIMENT<br>New Run	HMC_122.1	SRX1888309	76 m	ok	done	
RUN	HMC_123_000000000-A8DJ9_TCCCAGAACAAC_L001_R1_001.fastq.gz	SRR3732493	76 m	ok	done	2017-07-03 00:00:00
SAMPLE	HMC_123.1	SRS1531699	74 m	ok	done	

Type	Alias	Accession	Uploaded	Links	Files	Released
EXPERIMENT New Run	HMC_123.1	SRX1888310	76 m	ok	done	
RUN	HMC_124_000000000-A8DJ9_AGCTGGAAGTCC_L001_R2_001.fastq.gz	SRR3732494	76 m	ok	done	2017-07-03 00:00:00
SAMPLE	HMC_1225.1	SRS1531701	74 m	ok	done	
EXPERIMENT New Run	HMC_1225.1	SRX1888315	76 m	ok	done	
RUN	HMC_125_000000000-A8DJ9_CACGGTTGTGAG_L001_R2_001.fastq.gz	SRR3732495	76 m	ok	done	2017-07-03 00:00:00
SAMPLE	HMC_2284.1	SRS1531702	74 m	ok	done	
EXPERIMENT New Run	HMC_2284.1	SRX1888316	76 m	ok	done	
RUN	HMC_126_000000000-A8DJ9_GAGGAATAGCAG_L001_R1_001.fastq.gz	SRR3732496	76 m	ok	done	2017-07-03 00:00:00
SAMPLE	HMC_Mixed	SRS1531703	74 m	ok	done	
EXPERIMENT New Run	HMC_Mixed	SRX1888317	76 m	ok	done	
RUN	HMC_127_000000000-A8DJ9_ATCGGCGTTACA_L001_R1_001.fastq.gz	SRR3732497	76 m	ok	done	2017-07-03 00:00:00
SAMPLE	HMC_1	SRS1531704	74 m	ok	done	
EXPERIMENT New Run	HMC_1	SRX1888318	76 m	ok	done	
RUN	HMC_49_000000000-A8DJ9_AATCAGTCTCGT_L001_R1_001.fastq.gz	SRR3732498	76 m	ok	done	2017-07-03 00:00:00
SAMPLE	HMC_1.1	SRS1531705	74 m	ok	done	
EXPERIMENT New Run	HMC_1.1	SRX1888319	76 m	ok	done	
RUN	HMC_50_000000000-A8DJ9_CCTCGTTCGACT_L001_R2_001.fastq.gz	SRR3732499	76 m	ok	done	2017-07-03 00:00:00
SAMPLE	HMC_122	SRS1531706	74 m	ok	done	
EXPERIMENT New Run	HMC_122	SRX1888320	76 m	ok	done	
RUN	HMC_103_000000000-A8DJ9_TTCCTAGGTGAG_L001_R1_001.fastq.gz	SRR3732500	76 m	ok	done	2017-07-03 00:00:00
SAMPLE	HMC_2.1	SRS1531707	75 m	ok	done	
EXPERIMENT New Run	HMC_2.1	SRX1888322	76 m	ok	done	
RUN	HMC_52_000000000-A8DJ9_GACTTGGTATTC_L001_R1_001.fastq.gz	SRR3732502	76 m	ok	done	2017-07-03 00:00:00
SAMPLE	HMC_2	SRS1531708	75 m	ok	done	
EXPERIMENT New Run	HMC_2	SRX1888321	76 m	ok	done	
RUN	HMC_51_000000000-A8DJ9_AATCCGTACAGC_L001_R2_001.fastq.gz	SRR3732501	76 m	ok	done	2017-07-03 00:00:00
SAMPLE	HMC_3	SRS1531709	75 m	ok	done	
EXPERIMENT New Run	HMC_3	SRX1888323	76 m	ok	done	
RUN	HMC_53_000000000-A8DJ9_TACACGATCTAC_L001_R1_001.fastq.gz	SRR3732503	76 m	ok	done	2017-07-03 00:00:00
SAMPLE	HMC_3.1	SRS1531710	75 m	ok	done	

Type	Alias	Accession	Uploaded	Links	Files	Released
EXPERIMENT New Run	HMC_3.1	SRX1888324	76 m	ok	done	
RUN	HMC_54_000000000-A8DJ9_GCACACACGTTA_L001_R2_001.fastq.gz	SRR3732504	76 m	ok	done	**2017-07-03 00:00:00**
SAMPLE	HMC_12	SRS1531711	75 m	ok	done	
EXPERIMENT New Run	HMC_12	SRX1888326	76 m	ok	done	
RUN	HMC_56_000000000-A8DJ9_CAGGCGTATTGG_L001_R2_001.fastq.gz	SRR3732506	76 m	ok	done	**2017-07-03 00:00:00**
SAMPLE	HMC_6	SRS1531712	75 m	ok	done	
EXPERIMENT New Run	HMC_6	SRX1888325	76 m	ok	done	
RUN	HMC_55_000000000-A8DJ9_CACGCCATAATG_L001_R1_001.fastq.gz	SRR3732505	76 m	ok	done	**2017-07-03 00:00:00**
SAMPLE	HMC_17	SRS1531713	75 m	ok	done	
EXPERIMENT New Run	HMC_17	SRX1888327	76 m	ok	done	
RUN	HMC_57_000000000-A8DJ9_GGATCGCAGATC_L001_R1_001.fastq.gz	SRR3732507	76 m	ok	done	**2017-07-03 00:00:00**
SAMPLE	HMC_17.1	SRS1531714	75 m	ok	done	
EXPERIMENT New Run	HMC_17.1	SRX1888328	76 m	ok	done	
RUN	HMC_58_000000000-A8DJ9_GCTGATGAGCTG_L001_R2_001.fastq.gz	SRR3732508	76 m	ok	done	**2017-07-03 00:00:00**
SAMPLE	HMC_20	SRS1531715	75 m	ok	done	
EXPERIMENT New Run	HMC_20	SRX1888329	76 m	ok	done	
RUN	HMC_59_000000000-A8DJ9_AGCTGTTGTTTG_L001_R2_001.fastq.gz	SRR3732509	76 m	ok	done	**2017-07-03 00:00:00**
SAMPLE	HMC_20.1	SRS1531716	75 m	ok	done	
EXPERIMENT New Run	HMC_20.1	SRX1888330	76 m	ok	done	
RUN	HMC_60_000000000-A8DJ9_GGATGGTGTTGC_L001_R2_001.fastq.gz	SRR3732510	76 m	ok	done	**2017-07-03 00:00:00**
SAMPLE	HMC_123	SRS1531717	75 m	ok	done	
EXPERIMENT New Run	HMC_123	SRX1888331	76 m	ok	done	
RUN	HMC_104_000000000-A8DJ9_GCACGACAACAC_L001_R1_001.fastq.gz	SRR3732511	76 m	ok	done	**2017-07-03 00:00:00**
SAMPLE	HMC_24	SRS1531718	75 m	ok	done	
EXPERIMENT New Run	HMC_24	SRX1888332	76 m	ok	done	
RUN	HMC_61_000000000-A8DJ9_GCGATATATCGC_L001_R2_001.fastq.gz	SRR3732512	76 m	ok	done	**2017-07-03 00:00:00**
SAMPLE	HMC_24.1	SRS1531719	75 m	ok	done	
EXPERIMENT New Run	HMC_24.1	SRX1888333	76 m	ok	done	
RUN	HMC_62_000000000-A8DJ9_TAGGATTGCTCG_L001_R1_001.fastq.gz	SRR3732513	76 m	ok	done	**2017-07-03 00:00:00**
SAMPLE	HMC_27	SRS1531720	75 m	ok	done	

Type	Alias	Accession	Uploaded	Links	Files	Released
EXPERIMENT New Run	HMC_27	SRX1888334	76 m	ok	done	
RUN	HMC_63_000000000-A8DJ9_ATGTGCACGACT_L001_R2_001.fastq.gz	SRR3732514	76 m	ok	done	2017-07-03 00:00:00
SAMPLE	HMC_27.1	SRS1531721	75 m	ok	done	
EXPERIMENT New Run	HMC_27.1	SRX1888335	76 m	ok	done	
RUN	HMC_64_000000000-A8DJ9_ACGCGCAGATAC_L001_R1_001.fastq.gz	SRR3732515	76 m	ok	done	2017-07-03 00:00:00
SAMPLE	HMC_34	SRS1531722	75 m	ok	done	
EXPERIMENT New Run	HMC_34	SRX1888336	76 m	ok	done	
RUN	HMC_65_000000000-A8DJ9_GACTTTCCCTCG_L001_R2_001.fastq.gz	SRR3732516	76 m	ok	done	2017-07-03 00:00:00
SAMPLE	HMC_34.1	SRS1531723	75 m	ok	done	
EXPERIMENT New Run	HMC_34.1	SRX1888337	76 m	ok	done	
RUN	HMC_66_000000000-A8DJ9_ATCCCGAATTTG_L001_R2_001.fastq.gz	SRR3732517	76 m	ok	done	2017-07-03 00:00:00
SAMPLE	HMC_35	SRS1531724	75 m	ok	done	
EXPERIMENT New Run	HMC_35	SRX1888338	76 m	ok	done	
RUN	HMC_67_000000000-A8DJ9_GTTGGTCAATCT_L001_R2_001.fastq.gz	SRR3732518	76 m	ok	done	2017-07-03 00:00:00
SAMPLE	HMC_36	SRS1531725	75 m	ok	done	
EXPERIMENT New Run	HMC_36	SRX1888339	76 m	ok	done	
RUN	HMC_68_000000000-A8DJ9_TAGCTCGTAACT_L001_R1_001.fastq.gz	SRR3732519	76 m	ok	done	2017-07-03 00:00:00
SAMPLE	HMC_36.1	SRS1531726	75 m	ok	done	
EXPERIMENT New Run	HMC_36.1	SRX1888340	76 m	ok	done	
RUN	HMC_69_000000000-A8DJ9_CAGTGCATATGC_L001_R2_001.fastq.gz	SRR3732520	76 m	ok	done	2017-07-03 00:00:00
SAMPLE	HMC_37	SRS1531727	75 m	ok	done	
EXPERIMENT New Run	HMC_37	SRX1888341	76 m	ok	done	
RUN	HMC_70_000000000-A8DJ9_TCACGGGAGTTG_L001_R2_001.fastq.gz	SRR3732521	76 m	ok	done	2017-07-03 00:00:00
SAMPLE	HMC_1225	SRS1531728	75 m	ok	done	
EXPERIMENT New Run	HMC_1225	SRX1888342	76 m	ok	done	
RUN	HMC_105_000000000-A8DJ9_ATCGATCTGTGG_L001_R2_001.fastq.gz	SRR3732522	76 m	ok	done	2017-07-03 00:00:00
SAMPLE	HMC_38	SRS1531729	75 m	ok	done	
EXPERIMENT New Run	HMC_38	SRX1888343	76 m	ok	done	
RUN	HMC_71_000000000-A8DJ9_CTGCTAACGCAA_L001_R2_001.fastq.gz	SRR3732523	76 m	ok	done	2017-07-03 00:00:00
SAMPLE	HMC_39	SRS1531730	75 m	ok	done	

Type	Alias	Accession	Uploaded	Links	Files	Released
EXPERIMENT New Run	HMC_39	SRX1888344	75 m	ok	done	
RUN	HMC_72_000000000-A8DJ9_TTAGGGCTCGTA_L001_R1_001.fastq.gz	SRR3732524	75 m	ok	done	2017-07-03 00:00:00
SAMPLE	HMC_41	SRS1531731	75 m	ok	done	
EXPERIMENT New Run	HMC_41	SRX1888345	75 m	ok	done	
RUN	HMC_73_000000000-A8DJ9_TCTAGCGTAGTG_L001_R1_001.fastq.gz	SRR3732525	75 m	ok	done	2017-07-03 00:00:00
SAMPLE	HMC_41.1	SRS1531732	75 m	ok	done	
EXPERIMENT New Run	HMC_41.1	SRX1888346	75 m	ok	done	
RUN	HMC_74_000000000-A8DJ9_TCGAGGACTGCA_L001_R2_001.fastq.gz	SRR3732526	75 m	ok	done	2017-07-03 00:00:00
SAMPLE	HMC_42	SRS1531733	75 m	ok	done	
EXPERIMENT New Run	HMC_42	SRX1888347	75 m	ok	done	
RUN	HMC_75_000000000-A8DJ9_CGGAGCTATGGT_L001_R2_001.fastq.gz	SRR3732527	75 m	ok	done	2017-07-03 00:00:00
SAMPLE	HMC_43	SRS1531734	75 m	ok	done	
EXPERIMENT New Run	HMC_43	SRX1888348	75 m	ok	done	
RUN	HMC_76_000000000-A8DJ9_AAGAGATGTCGA_L001_R1_001.fastq.gz	SRR3732528	75 m	ok	done	2017-07-03 00:00:00
SAMPLE	HMC_44	SRS1531735	75 m	ok	done	
EXPERIMENT New Run	HMC_44	SRX1888350	75 m	ok	done	
RUN	HMC_78_000000000-A8DJ9_TACAGATGGCTC_L001_R2_001.fastq.gz	SRR3732530	75 m	ok	done	2017-07-03 00:00:00
SAMPLE	HMC_43.1	SRS1531736	75 m	ok	done	
EXPERIMENT New Run	HMC_43.1	SRX1888349	74 m	ok	done	
RUN	HMC_77_000000000-A8DJ9_TCCAAAGTGTTC_L001_R2_001.fastq.gz	SRR3732529	74 m	ok	done	2017-07-03 00:00:00
SAMPLE	HMC_46	SRS1531737	75 m	ok	done	
EXPERIMENT New Run	HMC_46	SRX1888351	75 m	ok	done	
RUN	HMC_79_000000000-A8DJ9_ACGTGTACCCAA_L001_R2_001.fastq.gz	SRR3732531	75 m	ok	done	2017-07-03 00:00:00
SAMPLE	HMC_46.1	SRS1531738	75 m	ok	done	
EXPERIMENT New Run	HMC_46.1	SRX1888352	75 m	ok	done	
RUN	HMC_80_000000000-A8DJ9_AAGGAGCGCCTT_L001_R2_001.fastq.gz	SRR3732532	75 m	ok	done	2017-07-03 00:00:00
SAMPLE	HMC_2284	SRS1531739	75 m	ok	done	
EXPERIMENT New Run	HMC_2284	SRX1888353	75 m	ok	done	
RUN	HMC_106_000000000-A8DJ9_CTTGTGTCGATA_L001_R2_001.fastq.gz	SRR3732533	75 m	ok	done	2017-07-03 00:00:00
SAMPLE	HMC_47	SRS1531740	75 m	ok	done	

Type	Alias	Accession	Uploaded	Links	Files	Released
EXPERIMENT New Run	HMC_47	SRX1888354	75 m	ok	done	
RUN	HMC_81_000000000-A8DJ9_CGATCCGTATTA_L001_R1_001.fastq.gz	SRR3732534	75 m	ok	done	2017-07-03 00:00:00
SAMPLE	HMC_48	SRS1531741	75 m	ok	done	
EXPERIMENT New Run	HMC_48	SRX1888355	75 m	ok	done	
RUN	HMC_82_000000000-A8DJ9_GTCTAATTCCGA_L001_R2_001.fastq.gz	SRR3732535	75 m	ok	done	2017-07-03 00:00:00
SAMPLE	HMC_49	SRS1531742	75 m	ok	done	
EXPERIMENT New Run	HMC_49	SRX1888356	75 m	ok	done	
RUN	HMC_83_000000000-A8DJ9_TCCGAATTCACA_L001_R2_001.fastq.gz	SRR3732536	75 m	ok	done	2017-07-03 00:00:00
SAMPLE	HMC_50	SRS1531744	75 m	ok	done	
EXPERIMENT New Run	HMC_50	SRX1888361	75 m	ok	done	
RUN	HMC_84_000000000-A8DJ9_ACGCCACGAATG_L001_R1_001.fastq.gz	SRR3732537	75 m	ok	done	2017-07-03 00:00:00
SAMPLE	HMC_50.1	SRS1531745	70 m	ok	done	
EXPERIMENT New Run	HMC_50.1	SRX1888362	74 m	ok	done	
RUN	HMC_85_000000000-A8DJ9_GGCCACGTAGTA_L001_R2_001.fastq.gz	SRR3732538	74 m	ok	done	2017-07-03 00:00:00
SAMPLE	HMC_101	SRS1531746	70 m	ok	done	
EXPERIMENT New Run	HMC_101	SRX1888363	74 m	ok	done	
RUN	HMC_86_000000000-A8DJ9_TAGGAACTGGCC_L001_R2_001.fastq.gz	SRR3732539	73 m	ok	done	2017-07-03 00:00:00
SAMPLE	HMC_102	SRS1531747	70 m	ok	done	
EXPERIMENT New Run	HMC_102	SRX1888364	74 m	ok	done	
RUN	HMC_87_000000000-A8DJ9_CTAGCGAACATC_L001_R2_001.fastq.gz	SRR3732540	74 m	ok	done	2017-07-03 00:00:00
SAMPLE	HMC_103	SRS1531748	70 m	ok	done	
EXPERIMENT New Run	HMC_103	SRX1888365	74 m	ok	done	
RUN	HMC_88_000000000-A8DJ9_GACAGGAGATAG_L001_R1_001.fastq.gz	SRR3732541	74 m	ok	done	2017-07-03 00:00:00
SAMPLE	HMC_104	SRS1531749	70 m	ok	done	
EXPERIMENT New Run	HMC_104	SRX1888366	74 m	ok	done	
RUN	HMC_89_000000000-A8DJ9_ATTCCTGTGAGT_L001_R2_001.fastq.gz	SRR3732542	74 m	ok	done	2017-07-03 00:00:00
SAMPLE	HMC_106	SRS1531750	70 m	ok	done	
EXPERIMENT New Run	HMC_106	SRX1888367	73 m	ok	done	
RUN	HMC_90_000000000-A8DJ9_GAGGCTCATCAT_L001_R1_001.fastq.gz	SRR3732543	73 m	ok	done	2017-07-03 00:00:00
SAMPLE	HMC_2485	SRS1531751	70 m	ok	done	

Type	Alias	Accession	Uploaded	Links	Files	Released
EXPERIMENT New Run	HMC_2485	SRX1888368	73 m	ok	done	
RUN	HMC_107_000000000-A8DJ9_TGAGCCGGAATC_L001_R1_001.fastq.gz	SRR3732544	73 m	ok	done	2017-07-03 00:00:00
SAMPLE	HMC_107	SRS1531752	70 m	ok	done	
EXPERIMENT New Run	HMC_107	SRX1888369	73 m	ok	done	
RUN	HMC_91_000000000-A8DJ9_TCCTCTGTCGAC_L001_R1_001.fastq.gz	SRR3732545	73 m	ok	done	2017-07-03 00:00:00
SAMPLE	HMC_108	SRS1531753	70 m	ok	done	
EXPERIMENT New Run	HMC_108	SRX1888370	73 m	ok	done	
RUN	HMC_92_000000000-A8DJ9_CTATTTGCGACA_L001_R2_001.fastq.gz	SRR3732546	73 m	ok	done	2017-07-03 00:00:00
SAMPLE	HMC_109	SRS1531754	70 m	ok	done	
EXPERIMENT New Run	HMC_109	SRX1888371	73 m	ok	done	
RUN	HMC_93_000000000-A8DJ9_AGTAGAGGGATG_L001_R1_001.fastq.gz	SRR3732547	73 m	ok	done	2017-07-03 00:00:00
SAMPLE	HMC_111	SRS1531755	70 m	ok	done	
EXPERIMENT New Run	HMC_111	SRX1888373	73 m	ok	done	
RUN	HMC_95_000000000-A8DJ9_AATGCCTCAACT_L001_R1_001.fastq.gz	SRR3732549	73 m	ok	done	2017-07-03 00:00:00
SAMPLE	HMC_110	SRS1531756	70 m	ok	done	
EXPERIMENT New Run	HMC_110	SRX1888372	73 m	ok	done	
RUN	HMC_94_000000000-A8DJ9_CGCAGCGGTATA_L001_R1_001.fastq.gz	SRR3732548	73 m	ok	done	2017-07-03 00:00:00
SAMPLE	HMC_112	SRS1531757	70 m	ok	done	
EXPERIMENT New Run	HMC_112	SRX1888374	73 m	ok	done	
RUN	HMC_96_000000000-A8DJ9_GGTGTCTATTGT_L001_R1_001.fastq.gz	SRR3732550	72 m	ok	done	2017-07-03 00:00:00
SAMPLE	HMC_113	SRS1531758	70 m	ok	done	
EXPERIMENT New Run	HMC_113	SRX1888375	73 m	ok	done	
RUN	HMC_97_000000000-A8DJ9_GTCAATTGACCG_L001_R2_001.fastq.gz	SRR3732551	73 m	ok	done	2017-07-03 00:00:00
SAMPLE	HMC_114	SRS1531759	70 m	ok	done	
EXPERIMENT New Run	HMC_114	SRX1888376	73 m	ok	done	
RUN	HMC_98_000000000-A8DJ9_ATGAGACTCCAC_L001_R1_001.fastq.gz	SRR3732552	73 m	ok	done	2017-07-03 00:00:00
SAMPLE	HMC_116	SRS1531760	70 m	ok	done	
EXPERIMENT New Run	HMC_116	SRX1888377	73 m	ok	done	
RUN	HMC_99_000000000-A8DJ9_GAATCTTCGAGC_L001_R1_001.fastq.gz	SRR3732553	73 m	ok	done	2017-07-03 00:00:00
SAMPLE	HMC_8619	SRS1531761	70 m	ok	done	

Type	Alias	Accession	Uploaded	Links	Files	Released
EXPERIMENT New Run	HMC_8619	SRX1888378	72 m	ok	done	
RUN	HMC_108_000000000-A8DJ9_GCGGCAATTACG_L001_R2_001.fastq.gz	SRR3732554	72 m	ok	done	**2017-07-03 00:00:00**
SAMPLE	HMC_101.1	SRS1531762	70 m	ok	done	
EXPERIMENT New Run	HMC_101.1	SRX1888379	72 m	ok	done	
RUN	HMC_109_000000000-A8DJ9_GAACTAGTCACC_L001_R1_001.fastq.gz	SRR3732555	72 m	ok	done	**2017-07-03 00:00:00**

New Experiment The SRA web submission interface for Sample creation has been replaced by the BioSample Submission Portal. Please make all sample submissions through the portal. SRA XML submissions are unchanged.

Set release date to: 2017-06-30 (YYYY-MM-DD)

Write to the Help Desk | Privacy Notice | Disclaimer | Accessibility Last update: Tue May 31 17:51:21 EDT 2016

National Center for Biotechnology Information | U.S. National Library of Medicine
